# Long-term autonomy, professional activities, cognition, and overall survival after awake functional-based surgery in patients with IDH-mutant grade 2 gliomas: a retrospective cohort study

**DOI:** 10.1016/j.lanepe.2024.101078

**Published:** 2024-09-20

**Authors:** Sam Ng, Valérie Rigau, Sylvie Moritz-Gasser, Catherine Gozé, Amélie Darlix, Guillaume Herbet, Hugues Duffau

**Affiliations:** a Department of Neurosurgery, Gui de Chauliac Hospital, Montpellier University Medical Center, 80 Av Augustin Fliche, 34295, Montpellier, France; b Institute of Functional Genomics, University of Montpellier, INSERM, CNRS, Team “Plasticity of Central Nervous System, Stem Cells and Glial Tumors,”, National Institute for Health and Medical Research (INSERM), U1191 Laboratory, 34091, Montpellier, France; c Department of Pathology and Onco-Biology, Gui de Chauliac Hospital, Montpellier University Medical Center, 80 Av Augustin Fliche, 34295, Montpellier, France; d University of Montpellier, 163 rue Broussonnet, 34000, Montpellier, France; e Department of Medical Oncology, Montpellier Regional Cancer Institute, 34298, Montpellier, France; f Praxiling Laboratory, UMR 5267, CNRS, Paul Valéry – Montpellier 3 University, rue de Mende, 34090, Montpellier, France; g Institut Universitaire de France, Paris, France

**Keywords:** WHO grade 2 gliomas, Awake surgery, Survival, Functional outcomes

## Abstract

**Background:**

In isocitrate dehydrogenase (IDH)-mutant low-grade gliomas (LGGs), awake functional-based resection (i.e., resection based on intraoperative functional responses rather than anatomical margins) has emerged as an efficient method to reduce tumour volume (TV) while minimizing postoperative deficits. Here, our goal was to assess the long-term onco-functional outcomes after awake functional-based resection in IDH-mutant LGGs, in conjunction with clinico-radiological and molecular factors.

**Methods:**

We retrospectively studied a consecutive cohort (June 1997–January 2023) of 949 patients. Six hundred patients with IDH-mutant LGGs benefited from an awake functional-based resection with a median follow-up of 7.8 years (95% Confidence interval [CI]: 7.1–8.4 years). The main outcomes were the overall survival (OS), the OS with Karnofsky performance status ≥80% (OS_KPS__≥__80%_), cognition measures, and professional activities at 12 months post-surgery.

**Findings:**

600 patients were included in the cohort (274 female [46.0%], median age: 36 years [Interquartile range, IQR: 30–44 years]). The rate of return to work was 93.7%. The impact of surgery on cognition was of limited magnitude. The median postsurgical TV of 2.5 mL (IQR: 0–8.0 mL). The median OS was over 20 years (median: NA, 95% CI: 17.0-NA years). The median OS_KPS__≥__80%_ was 14.7 years (95% CI: 13.2–17.2 years). Factors associated with longer OS and OS_KPS__≥__P80%_ were 1p19q codeletion (Hazard ratio [HR]_OS_: 0.27, 95% CI: 0.16–0.43, HR_KPS__≥__80%_:0.25, 95% CI: 0.17–0.36), supratotal resection (HR_OS_: 0.08, 95% CI: 0.005–0.40, HR_KPS__≥__80%_:0.12, 95% CI: 0.03–0.34) and total resection (HR_OS_: 0.31, 95% CI: 0.16–0.59, HR_KPS__≥__80%_:0.21, 95% CI: 0.12–0.36). Recursive partitioning analyses established three OS and OS_KPS__≥__80%_ prognostic groups, highlighting the contributions of histomolecular status, extent of resection, postsurgical and presurgical TV. Further propensity-matching analyses confirmed the oncological benefits of supratotal resections.

**Interpretation:**

Awake functional-based resection surgery in newly diagnosed IDH-mutant grade 2 LGG, was an effective strategy associated with long survival (median OS over 20 years) and long-term preservation of autonomy. More complete tumor resections favored better onco-functional outcomes across all molecularly-defined subtypes. Short-term effects were of limited magnitude regarding postoperative cognitive and professional outcomes. Supratotal functional-based resections offered additional survival benefits.

**Funding:**

None.

**Research in context**

**Evidence before this study**

Several retrospective cohort studies recently confirmed the positive impact of the extent of surgical resection on overall survival in molecularly defined isocitrate dehydrogenase (IDH) mutant low-grade gliomas. In this context, the concept of awake functional-based surgical resection (a surgical strategy whereby the surgeon decides to voluntarily abandon a tumour residue if critical brain areas subserving neurological functions and cognition are invaded, or conversely to remove a margin around the glioma core if functional networks are remote from the tumour) has progressively emerged in an attempt to optimize the extent of surgical resection while preserving patients’ quality of life. However, a systematic literature review resulting from a search in MEDLINE and Google Scholar (search terms and associated MeSH terms: [“glioma” OR “astrocytoma” OR “oligodendroglioma”] AND [“outcome” OR “cognition” OR “autonomy” OR “quality of life”] AND [“surgery” OR “awake surgery”]; search date: January 2013–December 2023) revealed that the long-term and integrated onco-functional results of awake functional-based surgical resection have not been investigated in large-scale cohorts of molecularly defined IDH-mutant low-grade gliomas.

**Added value of this study**

In this unique, large consecutive cohort, the authors investigated long-term oncological and functional results in 600 patients with IDH-mutant low-grade gliomas who received a similar, awake functional-based resection approach at diagnosis and a systematic follow-up every 6 months over a 25-year period of recruitment. Oncological variables (e.g., overall survival, histomolecular data, tumor volumes, extent of resection, adjuvant therapies) and functional variables (cognitive measures, professional activities at 12 months post-surgery, long-term Karnofsky Performance Status [KPS]) were analyzed to build onco-functional prognostic stratification models. For the first time to the authors’ knowledge, the contribution of awake functional-based resection in IDH-mutant low-grade gliomas was confirmed across all molecularly defined subtypes, both in terms of survival benefits and short/long-term preservation of an active life, with a median survival of 20 years and a median preservation of KPS ≥80% of 14.7 years after surgery.

**Implications of all the available evidence**

In patients eligible for surgery with IDH-mutant grade 2 gliomas, awake functional-based resection allows early resumption of cognition and professional activities and long survival with preservation of autonomy, regardless of molecular factors. In addition, supratotal functional-based resections offer additional survival benefits without compromising functional outcomes.

## Introduction

Low-grade gliomas (LGGs) are primary brain tumours that account for 15% of all gliomas, with an incidence rate of 1/100,000 person per year (French Brain Tumor Database).^1^ The impact of early and maximal surgical resection on the course of the disease has been firmly established in landmark studies demonstrating that surgical strategies offer a significant survival advantage in comparison to medical treatment alone.^2^ Yet, LGG classifications have progressively been revised over the past years and are now prominently based on histomolecular characteristics.^3^ Given the distinct clinical trajectory observed between molecularly defined subgroups of grade 2 LGGs, such as isocitrate dehydrogenase gene 1 or 2 (IDH)-mutant astrocytomas in comparison to IDH-mutant, 1p19q codeleted oligodendrogliomas, which present with more favorable spontaneous prognosis and a higher sensibility to chemotherapy agents,^4^ some authors suggest that maximal resection may not be a decisive therapeutic factor in this latter subtype of LGG.^5^

Besides, weighting the value of oncological and surgical therapies in confrontation with their potentially detrimental effects on patients' quality of life has become the cornerstone of modern neuro-oncological approaches based on the optimization of the “onco-functional balance”^6^ in patients who now have the opportunity to live longer. Admittedly, however, functional outcomes have scarcely been documented in surgical^7^ and non-surgical cohorts of LGGs with long-term follow-up, and current knowledge supports that a significant proportion of LGG patients may suffer from cognitive dysfunctions, and socio-professional disabilities.^8^

In this context, glioma surgery has made a huge step forward since the globalization of intraoperative awake functional mapping. Following this approach, the surgeon can decide to voluntarily abandon a tumour residue if critical cortico-subcortical areas subserving neurological functions and cognition are invaded, or conversely to remove a margin around the glioma core if functional networks are remote from the tumour.^9^ This surgical paradigm, namely “functional-based” resection surgery, is conceptually different from more classical surgical approaches aiming at resecting the tumour according to anatomical margins, with the support of various intraoperative technologies (e.g., fluorescence-guided surgery, or intraoperative MRI-guided surgery).^10^ Importantly, functional-based resection now serves as a gold-standard approach in the field of LGG surgery since it allows to significantly reduce the rate of language and motor postoperative deficit,^11^ as well as higher-order cognitive disturbances.^12^ However, although previous cohort analyses highlighted the potential effect of the extent of resection (EOR) on progression-free survival and overall survival (OS) under the molecular era,^13^, ^14^, ^15^ the long-lasting effects of functional-based resection on both survival and functional outcomes has never been investigated in conjunction with other major prognostic factors.

In this study, we intended to measure the long-lasting impact of awake functional-based surgical resection over the course of newly diagnosed IDH-mutant LGGs, along with other clinical, cognitive, radiological, histomolecular, and therapeutic factors. To this aim, we leveraged a unique monocentric cohort of 600 patients with IDH-mutant LGGs operated with systematic awake functional mapping between 1997 and 2023. We used a multi-step methodological approach to decipher factors associated with OS and long-term functional outcomes, using longitudinal Karnofsky performance status (KPS) metrics. Next, we applied recursive partitioning analyses, a statistical model validated in LGG^13^ to build innovative onco-functional risk stratification models combining known prognostic factors in the disease. Finally, to investigate the onco-functional outcomes related to total resections (i.e., complete removal of fluid-attenuated inversion recovery [FLAIR] abnormalities) and supratotal resections (i.e., complete removal of any FLAIR signal abnormalities [assessed on the postsurgical MRI] with a volume of the postoperative cavity larger than the presurgical FLAIR tumor volume [assessed on the presurgical MRI assessment]^16^), a propensity score matching analysis was used.

## Methods

### Study design and participants

In this retrospective cohort study, we aimed to model survival risk and long-term functional outcomes in patients with newly diagnosed grade 2 IDH-mutant LGGs. All participants provided informed consent. Eligible participants were patients consecutively operated on by the senior author (H.D.) for a suspected LGG between June 1997 and January 2023, with the following inclusion criteria: supratentorial grade 2 LGG (see Supplementary methods for more details regarding histomolecular data acquisitions), age ≥18 years, awake functional-based surgical resection, ≥3 months of follow-up. Radiological measures were blindly assessed by two observers. Details regarding volumetric calculations are reported in the Supplementary methods.

### Standard protocol approvals, registrations, and patient consents

The study was approved by an independent institutional review board of the ethical comity of research from the French National College of Neurosurgery (N°00011687 and N°00693) Relevant data were analyzed from a prospective databank (collection NEUROLOGIE DC-2013-2027). Written informed consent was obtained from the patients. Patients were not subjected to interventions outside the routine clinical management. The study conformed to the strengthening of the reporting of observational studies in epidemiology (STROBE) guidelines.

### Surgical technique

The same surgical technique was systematically used in all patients selected for analyses and was performed by the same neurosurgeon (H.D). Intraoperative functional mapping was performed through cortical and subcortical direct electrostimulation (DES) using an asleep-awake-asleep protocol. Briefly, following craniotomy under general anesthesia, the cortical surface was exposed, and the edges of the tumour were visualized by intraoperative ultrasound. Once the patient was awake, electrical cortical mapping was achieved with a bipolar electrode probe with a 5 mm inter-tip spacing (NIMBUS Stimulator, Newmedic, France), delivering a biphasic electric current (60 Hz, 1 ms pulse width, amplitude 1.50–3.50 mA). The amplitude was gradually increased until a positive response from the ventral premotor cortex was attained (i.e., transient speech articulatory disturbances). This amplitude was not modified during the remainder of the intraoperative mapping (including both cortical and subcortical axonal mapping). After completion of the cortical mapping, the tumour removal was performed by subpial dissection. Subcortical axonal DES mapping was performed to achieve tumour resection according to individual functional boundaries. Importantly, a stimulation site was considered functional if DES elicited disturbances at least three times in a nonconsecutive manner. Intraoperative monitoring of language, motor, and cognitive functions was achieved by a senior speech therapist and/or neuropsychologist who remained blinded to DES application. The following tasks could be used, depending on the location of the tumour: motor tasks, a picture naming task, a semantic association task (Pyramids and Palm Trees test), a dual-task, reading tasks, a mentalizing task (adapted version of the Read the mind in the eyes), a visual field monitoring tasks, a line bisection task, and a self-evaluation task.^17^

### Cohort characteristics

**Fig. 1:**
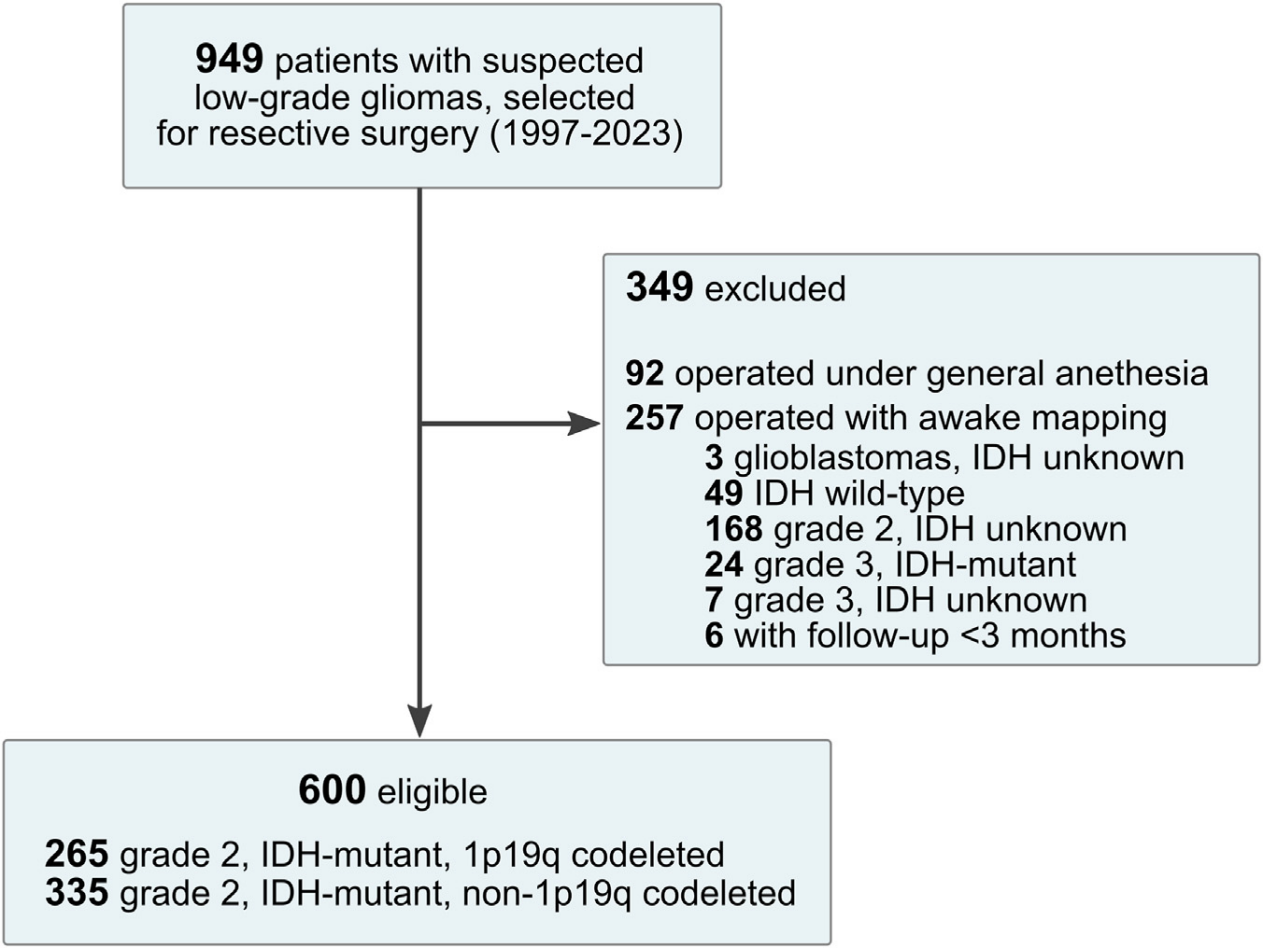
**Data flow diagram**. IDH indicates isocitrate dehydrogenase gene 1 or 2.

Clinical and radiological data were retrieved from 600 patients who underwent an awake functional-based resection from June 1997 to January 2023. The flow chart is provided in Fig. 1. Data collection ended in May 2023.

Resection subtypes were categorized as follows, to conform with previously published literature^18^: partial resection (postsurgical tumour volume [TV] >10 mL), subtotal resection (postsurgical TV > 0 mL and ≤10 mL), total resection (TR, defined by no postsurgical tumour residue on FLAIR-weighted MRI), and supratotal resection (supraTR, defined by no postsurgical tumour residue and additional resection margins beyond the presurgical pathological FLAIR signal). Adjuvant chemotherapy and radiotherapy were considered for subsequent analyses if they were delivered within 12 months following surgical resection.

Karnofsky performance status (KPS) was systematically assessed during the presurgical examination, the 3-month postsurgical examination, and every 6 months until the end of the follow-up or the death of the patient. The OS with KPS ≥80% metric was defined as the time between initial surgery and the first clinical examination objectifying a long-lasting (≥6 months) decrease in KPS under the score of 80% or death, whichever occurred first. Professional activities were systematically assessed during the presurgical examination and at 12 months post-surgery. Cognitive data were retrieved from two neuropsychological assessments (subset of 18 subtasks, see details in the Supplementary methods) performed by the same neuropsychologist and/or speech therapist 72 h before surgery and 3 months post-surgery. Of note, cognitive data were only obtained from French-speaking participants enrolled after 2013.

### Statistical analyses

All data were complete, apart from neuropsychological data. Data were analyzed from August 2023 to December 2023. Patients' demographic, clinical, radiological, and histomolecular characteristics were analyzed with descriptive statistics. Continuous variables were analyzed with two-tailed non-parametric Mann–Whitney U tests, given the non-Gaussian distribution of variables, as assessed with the Shapiro–Wilk test. Categorical variables were analyzed with Fisher's exact test and the Chi-square test when appropriate. Median follow-up was estimated with the reverse Kaplan–Meier method. All participants' cognitive scores were Z-transformed according to French normative data (taking into consideration educational level, age, and sex). In line with the standards in the neuropsychological literature, patients with a Z-score <−1.65 (score lower than 95% of the reference population) were considered to have a cognitive deficit.

OS was defined as the time between surgery and death. Progression-free survival (PFS) was defined as the time between surgery and tumor progression, based on the Neuro-Oncology assessment and RANO 2.0 criteria.^19^ Survival analyses were first conducted with the Kaplan–Meier method, and comparisons of survival curves were conducted with the log-rank test. Cox proportional hazard models (Cox-PHM) were used to assess associations between different variables and OS or OS with KPS ≥80%, both in univariable and multivariable settings. The proportionality of hazards was checked by inspection of the Schoenfeld residuals and log-minus-log survival plots. The linearity assumptions were checked by inspection of the deviance residuals vs covariate graph plots. The proportional hazard assumptions were not met for various univariable and multivariable models. As a result, several variables of interest could not be integrated into the Cox-PHM, including, among others, sex, EOR, presurgical TV, postsurgical TV, and the use of adjuvant radiotherapy.

To identify patient groups with different OS and OS with KPS ≥80% risks, we used recursive partitioning analyses (RPA) with the partDSA algorithm.^20^ This method was recently validated in LGGs^13^ and does not require the proportional hazard ratio assumptions. The Brier score was the chosen loss function for splitting and pruning. The tree minimizing the 5-fold cross-validated error as well as the most parsimonious tree within one standard error of the overall minimum error were selected. All known prognostic factors were included in the tree partitioning process, including age, sex, histomolecular status, EOR, tumour location, presurgical and postsurgical tumour volumes, adjuvant chemotherapy, adjuvant radiotherapy, presurgical and 3-month postsurgical KPS, and epileptic status. OS and OS with KPS ≥80% were then stratified by RPA risk groups and compared using Kaplan–Meier estimations with log-rank test, and univariable Cox-PHM.

Further, to estimate the impact of TR and supraTR, we computed a propensity score matching analysis. A caliper size of one-fourth of a standard deviation of the sample estimated propensity scores was applied. Matching was based on age, sex, histomolecular status, presurgical tumour volume, tumour location, chemotherapy, radiotherapy, presurgical and 3-month postsurgical KPS, and the rate of epilepsy/incidental findings before surgery. Characteristics of the matched subjects were systematically reviewed for comparisons. A 1:1 ratio between matched subjects was used to compare subjects with at least a total resection vs subjects without total resection (extent of resection <100%). Given the discrepancy between the number of patients with supratotal resection (extent of resection >100%) and control subjects, 1:1, 1:2, and 1:3 ratios were consecutively used to compare both groups. Similarly, 1:1 and 1:2 ratios analyses were consecutively used to compare supraTR and TR. The matching process was performed with the MatchIt package (https://cran.r-project.org/web/packages/MatchIt) and the optmatch package (https://cran.r-project.org/web/packages/optmatch). Graphical presentations were performed in Graphpad Prism 9.0 (https://www.graphpad.com) and Inkscape 1.1 (https://www.graphpad.com). All statistical analyses were conducted with R 4.3.2 (https://www.r-project.org).

### Role of the funding source

This study did not receive any funding or dedicated financial support.

## Results

Among the 600 patients who met eligibility, 276 (46.0%) were female, with a median age at surgery of 36 years (interquartile range [IQR]: 30–44 years). The series comprised 335 patients with IDH-mutant astrocytomas (55.8%) and 265 patients with IDH-mutant, 1p19q codeleted oligodendrogliomas (44.2%). Clinical and radiological characteristics, therapeutic factors, and descriptive comparisons between astrocytomas and oligodendrogliomas are summarized in Table 1. Details regarding incidental discoveries are provided in Supplementary Table S1. Of note, 464 patients (77.3%) were French and 136 were foreigners (22.6%) and came from 34 countries (details are provided in Supplementary Figure S1). Seventy-six patients (12.7%) first received a biopsy in another center, as their tumour was considered to be non-resectable. No patients died because of surgical complications. Four patients (0.7%) presented with a postoperative permanent deficit, on the basis of a standard clinical examination. Among 504 patients who had preoperative professional activities, 472 (93.7%) returned to work at 12 months postoperatively. The median presurgical TV was 45.0 (IQR: 22–86 mL). The median EOR was 94.0% (IQR: 87–100%) with a median residual TV of 2.5 mL (IQR: 0–8 mL). Z-transformed results from preoperative and 3-month postoperative neuropsychological tests are provided in Table 2. Before surgery, the rate of cognitive deficit (Z-score <−1.65) on a task-by-task basis ranged from 1.74% to 16.19%. After surgery, the rate of cognitive deficit on a task-by-task basis ranged from 2.18% to 22.79%, with significant performance declines in picture naming task (median of difference postoperative—preoperative = 0.00, W = −2298, p = 0.002), semantic fluency (median of difference postoperative—preoperative = −0.01, W = −11,924, p = 0.001), Stroop reading task (median of difference postoperative—preoperative = −0.33, W = −7230, p < 0.001) and episodic memory task (median of difference postoperative—preoperative = 0.00, W = −2591, p < 0.001) and significant performance improvements in Stroop inhibition task (median of difference postoperative – preoperative = 0.12, W = 3941, p = 0.021) and Stroop I-D task (median of difference postoperative—preoperative = 0.18, W = 5434, p = 0.002).

**Table 1:** Characteristics of the cohort.

Variables	Overall (n = 600)	IDH-mutant astrocytomas (n = 335)	IDH-mutant, 1p19q codeleted oligodendrogliomas (n = 265)	*p-value*
Age at surgery, years, median (IQR)	36 (30–44)	34 (29–41)	39 (32–47)	<0.0001^a^
*Sex*				
Female, n (%)	276 (46.0)	154 (46.0)	121 (45.7)	>0.999^b^
Male, n (%)	324 (54.0)	181 (54.0)	144 (54.3)	
*Tumor location*				
Left hemisphere, n (%)	353 (58.8)	209 (62.4)	144 (54.3)	0.065^b^
Right hemisphere, n (%)	241 (40.2)	124 (37.0)	117 (44.2)	
Bilateral, n (%)	6 (1.0)	2 (0.6)	4 (1.5)	
*Tumor volumes*				
Presurgical TV, mL, median (IQR)	45.0 (22.0–86.0)	50 (23–92)	40 (21.5–76)	0.127^a^
Postsurgical TV, mL, median (IQR)	2.5 (0.0–8.0)	2.5 (0.0–10.0)	3.0 (0.0–7.0)	0.716^a^
*Preoperative seizures*				
Yes, n (%)	482 (80.3)	266 (79.4)	216 (81.5)	0.537^b^
No, n (%)	118 (19.7)	69 (20.6)	49 (18.5)	
*Postoperative seizures*				
Long term (>3 months), n (%)	43 (8.8)	28 (8.4)	15 (5.7)	0.264^b^
Transient (<3 months), n (%)	54 (9.0)	29 (8.7)	25 (9.4)	0.775^b^
*Incidental findings*	112 (18.7)	65 (19.4)	47 (17.7)	0.673^b^
*Adjuvant chemotherapy*				
Yes, n (%)	46 (7.7)	28 (8.4)	18 (6.7)	0.538^b^
No, n (%)	554 (92.3)	307 (91.6)	247 (93.2)	
*Adjuvant radiotherapy*				
Yes, n (%)	9 (1.5)	4 (1.2)	5 (1.9)	0.518^b^
No, n (%)	591 (98.5)	331 (98.8)	260 (98.1)	
Extent of resection (median, IQR)	94.0 (87.0–100.0)	95.0 (88–100)	94.0 (87–100)	0.442^a^
*Type of resection*				
Supratotal, n (%)	49 (8.2)	27 (8.1)	22 (8.3)	>0.999^b^
Total, n (%)	134 (22.3)	80 (23.9)	54 (20.4)	0.325^b^
Subtotal, n (%)	303 (50.5)	155 (46.3)	148 (55.8)	0.021^b^
Partial, n (%)	114 (19.0)	73 (21.8)	41 (15.5)	0.059^b^
*Karnofsky performance status*				
Preoperative KPS (median, IQR)	100 (90–100)	100 (90–100)	100 (90–100)	0.430^a^
Postoperative KPS, 3 months (median, IQR)	90 (90–100)	90 (90–100)	90 (90–100)	0.423^a^
Postoperative deficit, n (%)	4 (0.7)	2 (0.6)	2 (0.8)	>0.999^b^
*Professional activities*				
Active before surgery, n (%)	504 (84.0)	289 (86.3)	215 (81.1)	0.094^b^
Active after surgery, n (%)	472 (78.7)	272 (81.2)	200 (75.5)	0.108^b^
*Deaths*				
Yes, n (%)	91 (15.2)	66 (19.7)	25 (9.4)	0.0005^b^
No, n (%)	509 (84.8)	269 (80.3)	240 (90.6)	
Unknown, n (%)	0 (0.0)	0 (0.0)	0 (0.0)	
Median follow-up, years (95% IC)	7.8 (7.1–8.4)	6.8 (6.1–7.5)	9.2 (8.3–9.6)	<0.0001^c^

a Two-tailed Mann–Whitney U test.

b Fisher's exact test.

c Log-rank test, reverse Kaplan–Meier method.

**Table 2:** Perioperative neurocognitive data.

Cognitive domain	Task	n	Mean Z-score (preoperative)	Mean Z-score (postoperative)	Median of differences	*p-value*^c^	Preoperative Z-score < −1.65, n (%)	Postoperative Z-score < −1.65, n (%)	*p-value*^d^
Language	Picture naming^a^	198	0.25 ± 0.8	−0.04 ± 1.4	0.00	0.002	9 (4.55)	17 (8.59)	0.154
Language/executive functions	Semantic fluency	324	−0.30 ± 1.1	−0.49 ± 1.1	−0.01	0.001	26 (8.02)	43 (13.27)	0.041
Language/executive functions	Phonological fluency	327	−0.30 ± 1.2	−0.34 ± 1.3	−0.15	0.493	35 (10.70)	46 (14.07)	0.235
Psychomotor speed/attention	TMT-A	229	0.44 ± 0.8	0.40 ± 0.7	0.00	0.547	4 (1.74)	5 (2.18)	>0.999
Executive functioning	TMT-B	226	0.18 ± 0.9	0.16 ± 1.0	0.05	0.509	7 (3.10)	12 (5.31)	0.349
Executive functioning	TMT B-A	221	−0.11 ± 0.9	−0.08 ± 1.1	0.11	0.131	10 (4.52)	20 (9.05)	0.087
Psychomotor speed/attention	Stroop naming	211	−0.24 ± 1.1	−0.35 ± 1.5	0.00	0.492	21 (9.95)	30 (14.22)	0.232
Psychomotor speed/attention	Stroop reading	215	−0.41 ± 1.5	−0.72 ± 1.5	−0.33	<0.001	32 (14.88)	49 (22.79)	0.048
Executive functioning	Stroop I	215	−0.24 ± 1.4	−0.18 ± 1.5	0.12	0.021	20 (9.30)	23 (10.70)	0.748
Executive functioning	Stroop I-D	211	−0.21 ± 1.4	−0.06 ± 1.3	0.18	0.002	17 (8.06)	21 (9.95)	0.610
Semantics	PPTT^a^	210	−0.46 ± 1.5	−0.26 ± 1.5	0.00	0.078	34 (16.19)	26 (12.38)	0.329
Working memory	Forward span	203	−0.05 ± 1.1	0.06 ± 1.1	0.00	0.221	16 (7.88)	11 (5.42)	0.426
Working memory	Backward span	203	0.08 ± 1.1	0.14 ± 1.0	0.00	0.325	5 (2.46)	3 (1.48)	0.724
Verbal episodic memory	Free recall (RL-RI-16)	155	−0.50 ± 0.9	−0.60 ± 1.3	0.14	0.534	14 (9.03)	28 (18.06)	0.030
Verbal episodic memory	Total recall (RL-RI-16)	150	−0.35 ± 0.6	−0.60 ± 0.7	0.00	<0.001	6 (4.00)	23 (13.33)	0.001
Visuospatial functions	Bell test (total omissions)^b^	155	−0.05 ± 1.2	0.03 ± 1.1	0.00	0.405	15 (9.68)	10 (7.10)	0.405
Visuospatial functions/Non-verbal episodic memory	Rey/Taylor figure (immediate)^b^	65	0.20 ± 1.1	−0.05 ± 1.2	−0.20	0.199	4 (6.15)	8 (12.31)	0.364
Visuospatial functions/Non-verbal episodic memory	Rey/Taylor figure (delayed)^b^	65	0.27 ± 1.1	−0.02 ± 1.2	−0.20	0.244	3 (4.62)	6 (9.23)	0.492

Preoperative neuropsychological assessment was performed in the 72 h before the surgery.

Postoperative neuropsychological assessment was performed at 3 months postoperatively, by the same neuropsychologist and/or speech therapist.

All participants' raw scores were appropriately aligned to published French normative data (adjusted according to educational level, age, and sex) and subsequently converted into z-scores.

Picture naming scores were obtained with the DO80: Dénomination orale d'images, PPTT: Pyramid and palm tree test, TMT: Trail making test, R&T Rey or Complex Figure.

All participants' raw scores were appropriately aligned to published French normative data (adjusted according to educational level, age, and sex) and subsequently converted into z-scores.

The neuropsychological battery is detailed in the Supplementary files.

a Only patients with left-sided tumours.

b Only patients with right-sided tumours.

c Wilcoxon matched-pairs signed rank test, two-tailed *p-value*, uncorrected.

d Fisher's exact test, two-tailed *p-value*, uncorrected.

**Fig. 2:**
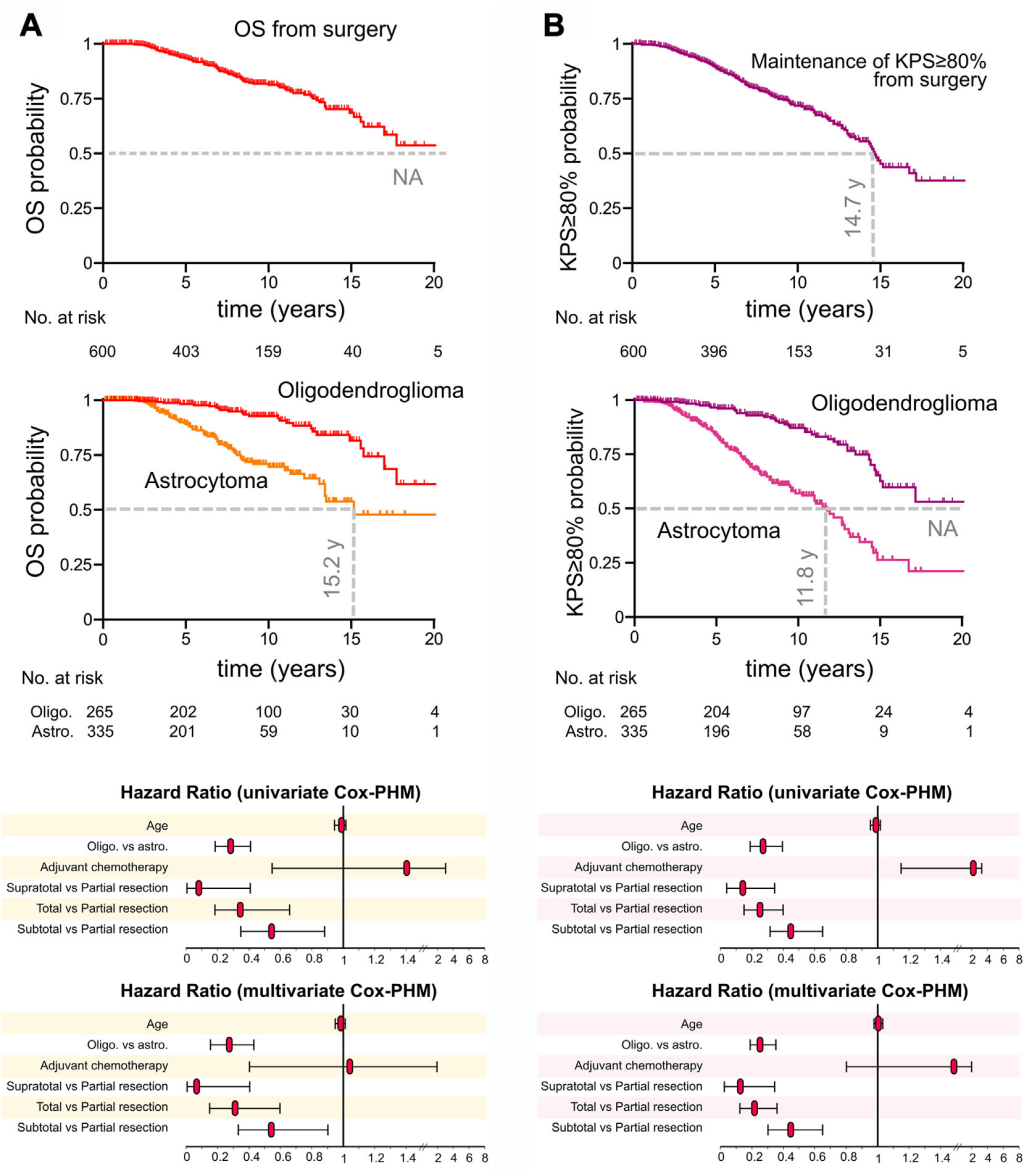
**Kaplan–Meier curves and Hazard ratios for overall survival and overall survival with Karnofsky performance status ≥80%**. A., from top to bottom, Kaplan–Meier curves for overall survival from initial surgery in all patients (n = 600), in IDH-mutant astrocytomas (n = 335) vs IDH-mutant 1p19q codeleted oligodendrogliomas (n = 265), and Hazard ratio results for overall survival (n = 600) using univariate Cox proportional hazard models and multivariate Cox proportional hazard models in variables eligible for analyses. B., from top to bottom, Kaplan–Meier curves for overall survival with Karnofsky performance status ≥80% from initial surgery in all patients (n = 600), in IDH-mutant astrocytomas (n = 335) vs IDH-mutant 1p19q codeleted oligodendrogliomas (n = 265), and Hazard ratio results for overall survival with Karnofsky performance status ≥80% (n = 600) using univariate Cox proportional hazard models and multivariate Cox proportional hazard models in variables eligible for analyses. Astro., IDH-mutant astrocytoma; Cox-PHM, Cox proportional hazard model; KPS, Karnofsky performance status; NA, not available; Oligo., IDH-mutant; 1p19q codeleted oligodendrogliomas; OS, overall survival.

Survival results are provided in Fig. 2 and in the Supplementary Tables S2–S5. As of May 2023, the median follow-up was 7.8 years (95% Confidence Interval [CI]: 7.1–8.4 years), and the median OS was over 20 years (95% CI: 17.0-NA years, Fig. 2A). The median OS with KPS ≥80% was 14.7 years (95% CI: 13.2–17.2 years, Fig. 2B). The median PFS was 8.3 years (95% CI: 7.9–8.8 years). The OS was significantly longer in oligodendrogliomas than in astrocytomas (median survival: NA [95% CI: 17.8-NA years] vs 15.2 years [95% CI: 13.4-NA years], respectively, p < 0.0001, log-rank test), along with OS with KPS ≥80% (median survival: NA [95% CI: 15.0-NA years] vs 11.8 [95% CI: 9.8–12.9 years], respectively, p < 0.0001, log-rank test). In both univariable and multivariable Cox-PHM, oligodendroglioma subgroup (multivariable HR: 0.27, 95% CI: 0.16–0.43, p < 0.0001), supraTR (multivariable HR: 0.08, 95% CI: 0.005–0.40, p = 0.016), TR (multivariable HR: 0.31, 95% CI: 0.16–0.59, p = 0.0005) and subtotal resections (multivariable HR: 0.54, 95% CI: 0.34–0.90, p = 0.014) were significantly associated with longer OS. Regarding OS with KPS ≥80%, univariable and multivariable models indicated positive effects for oligodendroglioma subgroup (multivariable HR: 0.25, 95% CI: 0.17–0.36, p < 0.0001), supraTR (multivariable HR: 0.12, 95% CI: 0.03–0.34, p = 0.0005), TR (multivariable HR: 0.21, 95% CI: 0.12–0.36, p < 0.0001) and subtotal resection (multivariable HR: 0.45, 95% CI: 0.31–0.66, p < 0.0001). Adjuvant chemotherapy was associated with shorter OS with KPS ≥80% in univariable models, but not in multivariable models (multivariable HR: 1.56, 95% CI: 0.85–2.65, p = 0.122). Additional PFS results are illustrated in Supplementary Figures S2A and B).

**Fig. 3:**
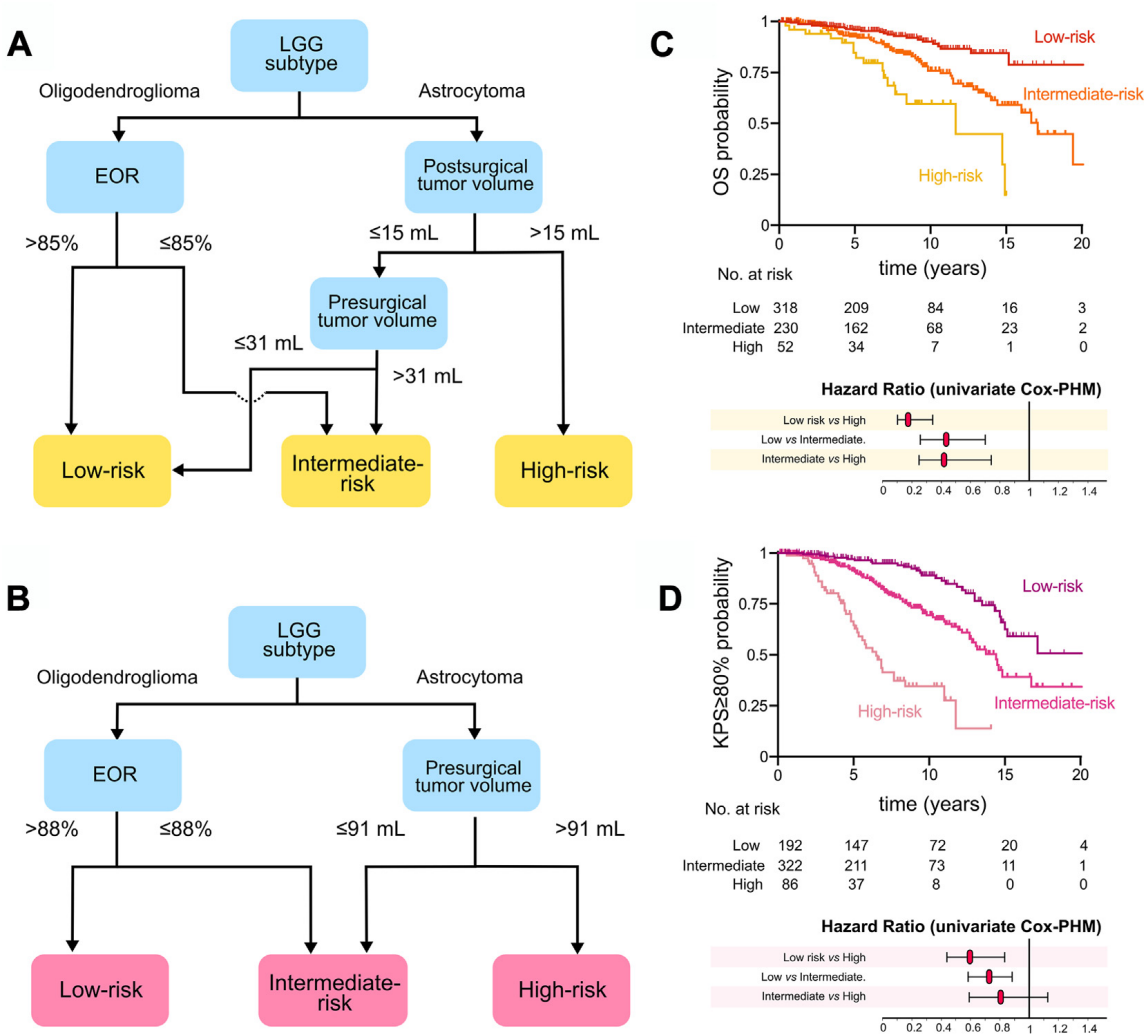
**Recursive partitioning analysis for overall survival, overall survival with Karnofsky performance status ≥80% and resulting Kaplan–Meier curves and Hazard ratios stratified by risk groups**. A., Three risk groups were determined by recursive partitioning analysis (n = 600) for overall survival, based on the following factors: age at surgery, sex, histomolecular status, the extent of resection, tumour location, presurgical and postsurgical tumour volumes, adjuvant chemotherapy, adjuvant radiotherapy, presurgical and 3-month postsurgical Karnofsky performance status, and epileptic status. B., Three risk groups were determined by recursive partitioning analysis (n = 600) for overall survival with Karnofsky performance status ≥80%, based on the following factors: age at surgery, sex, histomolecular status, the extent of resection, tumour location, presurgical and postsurgical tumour volumes, adjuvant chemotherapy, adjuvant radiotherapy, presurgical and 3-month postsurgical Karnofsky performance status, and epileptic status. C., Kaplan–Meier curves and hazard ratios for overall survival stratified by risk groups, as determined by recursive partitioning analyses. D., Kaplan–Meier curves and hazard ratios for overall survival with Karnofsky performance status ≥80%, stratified by risk groups, as determined by recursive partitioning analyses. Cox-PHM, Cox proportional hazard model; EOR, extent of resection; KPS, Karnofsky performance status; LGG, low-grade glioma; OS, overall survival.

In addition, because the proportional hazard assumptions were not met for several univariable and multivariable models, we used RPA models. Three survival risk groups were determined, both for OS and OS with KPS ≥80% (characteristics of these groups are detailed in Supplementary Tables S6–S9). Regarding OS risk stratification (Fig. 3A), a low-risk group with longer OS (n = 318, median survival: NA, 95% CI: 20.2-NA years) included oligodendrogliomas with EOR >85% and astrocytomas with postsurgical TV ≤15 mL and presurgical TV ≤31 mL. An intermediate-risk group (n = 230, median survival: 17.1 years, 95% CI: 14.4-NA years) was composed of oligodendrogliomas with EOR ≤85% and astrocytomas with postsurgical TV ≤15 mL and presurgical TV >31 mL. Finally, a high-risk group (n = 52, median survival: 11.7 years, 95% CI: 7.2–14.9 years) comprised patients with astrocytomas and postsurgical TV >15 mL. Regarding OS with KPS ≥80% risk stratification (Fig. 3B), a low-risk group with longer OS with KPS ≥80% (n = 192, median survival: NA, 95% CI: 15.0-NA years) comprised patients with oligodendrogliomas and EOR >88%. An intermediate-risk group (n = 322, median survival: 14.4 years, 95% CI: 12.7–16.8 years) combined oligodendrogliomas with EOR ≤88% and astrocytomas with presurgical TV ≤91 mL, while a high-risk group (n = 86, median survival: 6.5 years, 95% CI: 5.1–7.7 years) included patients with astrocytomas and presurgical TV >91 mL. Survival curves from distinct survival risk groups were statistically different (log-rank test, p < 0.0001, both for OS and OS with KPS ≥80% risk stratification, Fig. 3C and D).

**Fig. 4:**
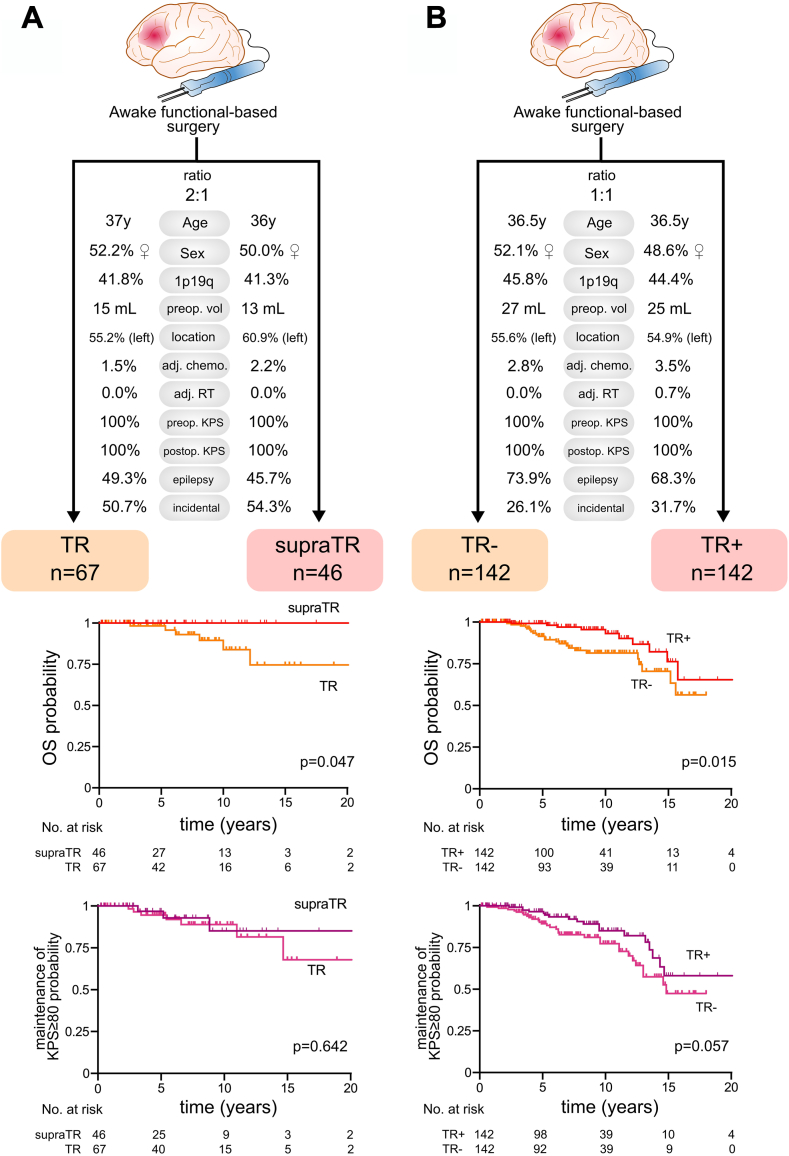
**Propensity score analysis of overall survival and overall survival with Karnofsky performance status ≥80% in patients with supratotal resection (supraTR) vs patients with total resection (TR) and in patients with total resection or more (TR+) vs patients with less than total resection (TR−)**. A., Characteristics of patients selected for comparison with propensity score matching (supratotal resection vs total resection). Given the discrepancy between the number of control subjects (TR, n = 134) and the number of patients with supratotal resections (supraTR, n = 49), a 2:1 ratio was applied. Of note, 1:1 ratio analysis indicated non-significant difference between survival curves, although the same trend was observed (Log-rank test, p = 0.062, see Supplements). Kaplan–Meier curves for overall survival and overall survival with Karnofsky performance status ≥80% stratified by resection group are presented below. Log-rank tests were used for statistical comparisons. B., Characteristics of patients selected for comparison with propensity score matching (at least total resection or TR+ vs less than total resection or TR−). Kaplan–Meier curves for overall survival and overall survival with Karnofsky performance status ≥80% stratified by resection group are presented below. Log-rank tests were used for statistical comparisons.

Further, to investigate the impact of supraTR and TR on OS and OS with KPS ≥80%, we used propensity score matching analyses. The characteristics of the matched cohorts selected for statistical comparisons are presented in Fig. 4 and are detailed, together with the summary of the balance of matched data in the Supplementary Tables S10–S14. A longer OS was observed in patients who underwent supraTR vs patients who did not undergo supraTR (p = 0.041 by log-rank test. We repeated the matching process with a ratio of 1:1, 1:2, and 1:3 patients and consistently found the same results (see Supplementary Figures S3–S5). Patients who underwent a supraTR tended to benefit from longer OS than patients with TR in a 1:1 ratio comparison (p = 0.062, Supplementary Figure S6). Further, a 1:2 ratio matching analysis resulted in a longer OS in patients with supraTR in comparison to patients with TR (p = 0.047 by long-rank test, Fig. 4A). No difference was observed in terms of OS with KPS ≥80%, and PFS (see Supplementary Figures S7 and S8). Similarly, patients who underwent at least a TR (TR + group) had longer OS than other patients (p = 0.015 by log-rank test) and tended to benefit from longer OS with KPS ≥80% (p = 0.057 by log-rank test, Fig. 4B). Additional analyses comparing the OS impact of supraTR vs TR in patients with preoperative TV >20 mL, >30 mL, and >40 mL were not conclusive (Supplementary Figure S9).

## Discussion

In this study, we capitalized on a large consecutive cohort with IDH-mutant LGGs to establish the long-lasting onco-functional results provided by awake functional-based resection surgery, among other known prognostic factors, including molecular, therapeutic, and clinical parameters. This is the first experience reporting a median OS over 20 years with 93.7% of return to work at 12 months post-surgery and a median OS with KPS ≥80% of 14.7 years. Our findings support the significant impact of molecular features, EOR, postsurgical TV, and presurgical TV on both OS and the long-term maintenance of functional autonomy. The effect of awake functional-based surgery on OS was confirmed in IDH-mutant astrocytomas and IDH-mutant 1p19q codeleted oligodendrogliomas, supporting previous hypotheses drawn from the pre-molecular era. In addition, few patients presented with debilitating functional impairments after surgery (<1% of patients suffered from permanent neurological deficits), and EOR was found to be an independent prognostic factor of long-term preservation of autonomy, as assessed with longitudinal KPS evaluations. Finally, the OS advantage of gross total resection and supratotal resection was validated through propensity score matching analyses, although no effects were observed regarding the long-term preservation of patients’ autonomy.

Using standard Cox-PHM, we investigated the effects of various spontaneous and therapeutic factors on OS. Both histomolecular features and the type of surgical resection appeared to be determinant prognostic variables, as already suggested by several studies.^14^^,^^15^^,^^21^^,^^22^ Next, we replicated the methods recently developed by Hervey-Jumper et al.,^13^ using RPA to fully integrate all known prognostic variables. Our findings regarding survival analyses were close but not strictly identical to those reported in this study. Importantly, the histomolecular status (based on 1p19q co-deletion) was systematically found as a preponderant prognostic factor delineating the first node of risk stratification. However, patients with astrocytomas presenting with low postsurgical TV (≤15 mL) and low presurgical TV (≤31 mL) mirrored the long-term survival trajectory of oligodendrogliomas with a large extent of resection (>85%). Conversely, oligodendrogliomas with EOR ≤85% had similar prognostic risks as astrocytomas with low postsurgical TV (≤15 mL) and high presurgical TV (>31 mL), defining an intermediate prognostic risk group. Finally, patients with the worst survival prognosis were astrocytomas with postsurgical TV >15 mL. These findings confirm the major impact of the EOR across all subtypes of IDH-mutant LGGs. Furthermore, the survival advantage of supratotal resection and total resection was suggested as an independent prognostic factor. Such results tend to confirm the recent observations that EOR greater than 75% is an independent therapeutic factor,^13^ and bring additional evidence regarding the role of supratotal resection in IDH-mutant LGGs compared to total resection, beyond its already known impact on progression-free survival.^23^

Another strength of this study is to provide critical insight into the long-term functional outcomes of LGG patients who benefitted from a homogeneous surgical approach relying on intraoperative functional responses to determine the extent of tumoural resection. Functional outcomes have been limitedly reported in the literature, and previous studies indicate detrimental effects of LGG diagnosis and related oncological therapeutics in terms of working abilities,^24^ wellbeing,^25^ and cognitive functioning.^26^ Strikingly, our results indicate that most patients being candidates for awake functional-based resection can overcome this poor functional prognosis early after surgery with a high rate of return to work at 12 months, which seems to be consistent with the favorable neurocognitive outcomes reported at 3 months postoperatively.^12^ On the other hand, the median maintenance of KPS ≥80% reached 14.7 years after initial surgery across all LGG subtypes. To investigate factors conditioning long-term functional outcomes, we also combined Cox-PHM analyses and RPA. Functional outcomes were significantly impacted by the histomolecular status (oligodendroglioma had a better functional prognosis than astrocytomas), the EOR, and the presurgical TV. Interestingly, the effect of surgery was particularly crucial in oligodendrogliomas, with a better functional prognosis following an EOR >88%, whereas the functional prognosis of astrocytoma was mostly driven by the presurgical TV.

Taken together, the findings reported in this study provide a novel overview of both long-term oncological and functional outcomes in IDH-mutant LGGs and grant a comprehensive onco-functional risk stratification model that may assist oncologists in their decision for early adjuvant treatment options vs delayed treatment strategies. It is worth mentioning that such a model, which integrates a wide range of clinical, radiological, and therapeutic factors is still lacking to inform postoperative neuro-oncological strategies in IDH-mutant LGGs. Therefore, some clinicians keep on employing the historical dichotomy between “low-risk” and “high-risk” gliomas to administer adjuvant oncological strategies.^27^ Beyond the beneficial effects of these adjuvant therapies,^28^^,^^29^ early postoperative administrations of chemo-radiotherapy may result in over-treatments and detrimental side effects in the long run, such as neurocognitive declines a few years after radiotherapy^30^ or deleterious hypermutations following temozolomide administration. Crucially, although this topic has raised several debates within the neuro-oncological community over the last decade, it now becomes even more relevant with the rapid emergence of anti-IDH therapies, which will urge clinicians to rethink again their adjuvant armamentarium following initial surgery.^31^

This study has several limitations. First, participants selected for analysis were patients with resectable LGGs, as determined by the treating neurosurgeon. Therefore, our findings cannot be generalized to patients with non-resectable gliomas who only benefited from a biopsy, although it is worth mentioning that 16.7% of patients in the current cohort were preliminarily presumed to carry non-resectable tumours and underwent a biopsy before being addressed to our medical center. Second, our study does not include an external validation cohort analysis. To the authors’ knowledge, there is, however, no other cohort of LGGs with long-term follow-up providing longitudinal functional evaluations that could offer the opportunity for an external test set. Third, even though the KPS reflects patients' global autonomy, it only partially and indirectly represents health-related quality of life. Previous studies have reported detailed long-term health-related quality-of-life outcomes in a subset of LGG patients, including a decrease in general health perception, decreases in physical functioning subscales,^32^ and persistent depression and fatigue.^25^^,^^33^ Such health-related quality-of-life investigations could not be assessed longitudinally in the present study due to the retrospective nature of data analyses. Fourth, although it is known that oncological strategies largely differ across institutions, the strategies applied in our institution may not be representative of the standard of care, especially regarding the limited use of early postoperative adjuvant oncological therapies (7.7% of patients received adjuvant chemotherapy, 1.5% of patients received adjuvant radiotherapy). Finally, our analyses may not capture therapeutic interactions resulting from delayed or repeated oncological treatment over time, which is beyond the scope of this study.

In conclusion, maximal awake functional-based resection surgery in newly diagnosed IDH-mutant grade 2 LGG, was an effective strategy associated with long survival (median OS over 20 years) and long-term preservation of autonomy (median OS with KPS ≥80% of 14.7 years). More complete tumor resections favored better onco-functional outcomes across all molecularly defined subtypes. Short-term effects were of limited magnitude regarding postoperative neurocognitive outcomes and work resumption. Onco-functional prognostic stratification models supported the impact of histomolecular status, extent of resection, postsurgical tumour volume, and presurgical tumour volume. In addition, supratotal and total functional-based resections offered additional survival benefits, without compromising functional outcomes.

## Contributors

HD contributed to the study concept. SN and HD designed, analyzed, and drafted the manuscript. VR, SMG, CG, AD, GH, and HD acquired data. All authors (SN, VR, SMG, CG, AD, GH and HD) had insight into the data and critically reviewed and interpreted the data. HD supervised the study. All authors read and approved the final draft of the manuscript and had final responsibility for the decision to submit for publication.

## Data sharing statement

Anonymized data not published within this article will be made available by request from any qualified investigator.

## Declaration of interests

All authors declare no relevant conflict of interests.

## Appendix A Supplementary data

Supplementary Content
