# Supplementary material for: Long-term autonomy, professional activities, cognition, and overall survival after awake functional-based surgery in patients with IDH-mutant grade 2 gliomas: a retrospective cohort study
Source: Lancet Reg Health Eur. 2024 Sep 20;46:101078. doi: 10.1016/j.lanepe.2024.101078 (PMC11458993; doi:10.1016/j.lanepe.2024.101078)
Supplement: Supplementary Content [file mmc1.docx]

**Supplementary materials**

**Long-term autonomy, professional activities, cognition, and overall survival after awake functional-based surgery in patients with IDH-mutant grade 2 gliomas: a retrospective cohort study**

Sam Ng,^1,2^ Valérie Rigau,^2,3^ Sylvie Moritz-Gasser,^1,2,4^ Catherine Gozé,^2,3^ Amélie Darlix,^2,5^ Guillaume Herbet,^1,4,6,7^ and Hugues Duffau^1,2^

^1^Department of Neurosurgery, Gui de Chauliac Hospital, Montpellier University Medical Center, 80 Av Augustin Fliche, 34295 Montpellier, France

^2^Institute of Functional Genomics, University of Montpellier, INSERM, CNRS, Team “Plasticity of Central Nervous System, Stem Cells and Glial Tumors,” National Institute for Health and Medical Research (INSERM), U1191 Laboratory, 34091 Montpellier, France

^3^Department of Pathology and Onco-biology, Gui de Chauliac Hospital, Montpellier University Medical Center, 80 Av Augustin Fliche, 34295 Montpellier, France

^4^University of Montpellier, 163 rue Broussonnet, 34000 Montpellier, France

^5^Department of Medical Oncology, Montpellier regional Cancer Institute, 34298 Montpellier, France (Amélie Darlix)

^6^Praxiling laboratory, UMR 5267, CNRS, Paul Valéry – Montpellier 3 University, rue de Mende, 34090 Montpellier, France

^7^Institut Universitaire de France, Paris, France

*Corresponding author

Dr Sam Ng, M.D.

Department of Neurosurgery, Gui de Chauliac Hospital, Montpellier University Medical Center, 80 Avenue Augustin Fliche, 34295 Montpellier, France

Phone: +33 4 67 33 66 12; Fax: +33 4 67 33 69 12; Email: s-ng@chu-montpellier.fr

1. **Supplementary methods**
2. **Supplementary tables**
3. **Supplementary figures**

**Supplementary Methods 1.** Details on radiological data acquisitions

Tumors were segmented manually on FLAIR-weighted MRI to compute the volumes. In the first period of the series (1997-2008), an estimation of these volumes was calculated by the ellipsoid approximation (D1×D2×D3/2). Since 2008, a dedicated software (Myrian, Intrasense, France) has been used for segmentation. Both methods (ellipsoid approximations and computed volumetry) were assessed by two blinded observers (S.N. and H.D.).

Presurgical tumor volumes were determined on the presurgical MRIs, performed in the 72h before surgery. Postsurgical tumor volumes were computed on the 3-month postoperative MRIs, to avoid artifacts related to early postsurgical MRI acquisition (e.g., blood signals and tissue deformations).

The preoperative tumor volumes estimated by ellipsoid approximation method (55.41 ± 45.70 mL, n=54) were not different from the preoperative tumor volumes computed with segmentation volumetry (57.99 ± 45.94 mL, n=546; two-tailed t-test, t=0.3944, df=598, p=0.693).

The postoperative tumor volumes estimated by ellipsoid approximation method (8.44 ± 27.75 mL, n=54) were not different from the preoperative tumor volumes computed with segmentation volumetry (6.42 ± 10.91 mL, n=546; two-tailed t-test, t=1.066, df=598, p=0.287).


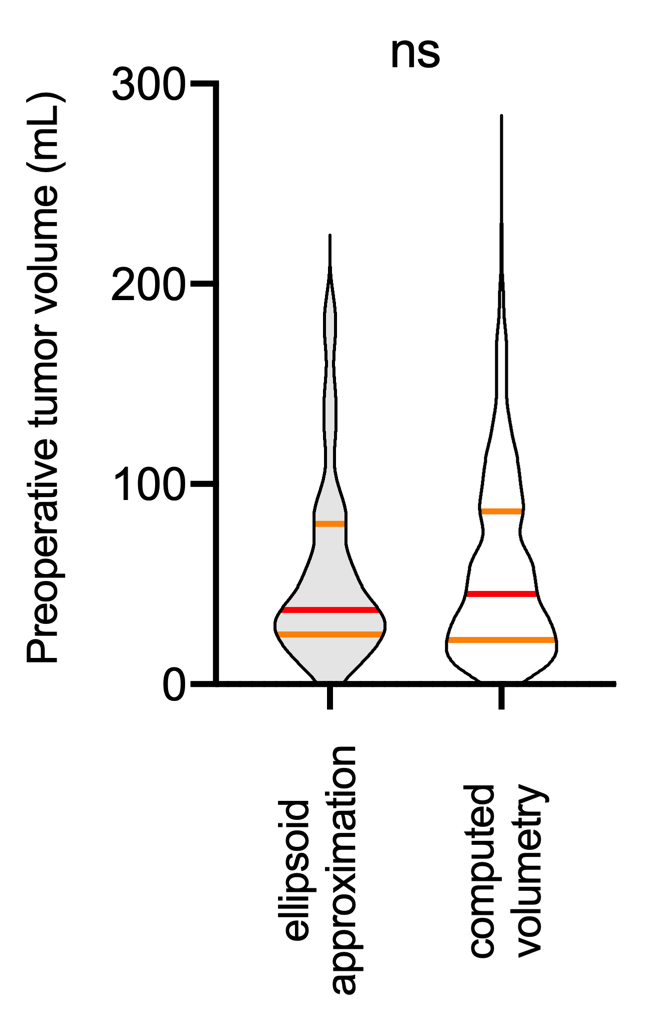

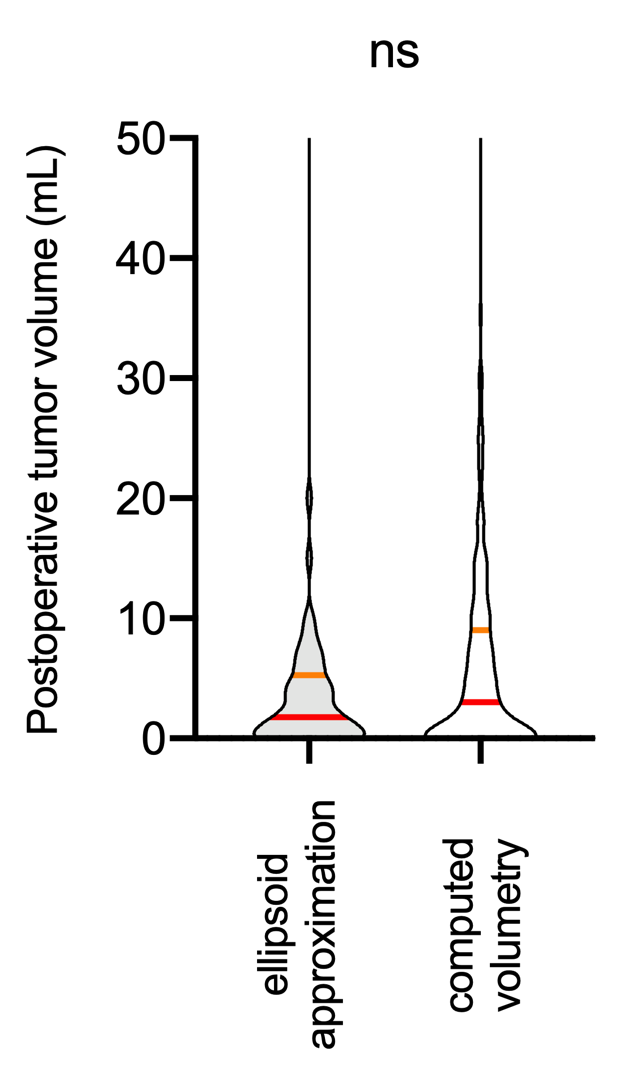


**Supplementary Methods 2.** Details on molecular data acquisitions

Immunohistochemistry for IDH1 R132H was systematically used. Between 2010 and 2018, the determination of IDH gene mutation status was made by direct Sanger sequencing. Since 2018, mutations in IDH genes have been sought as part of a wider panel of next-generation sequencing (NGS) analyzed genes (French Institut National du Cancer “INCa” panel, second edition between 2018 and 2019, then third edition since 2019).

The 1p19q codeletion was systematically investigated in low-grade gliomas since 2007. From 2007 to 2017 a Loss-of-heterozygosity assay (LOH) with 12 microsatellite markers was performed. Since 2017 all Copy Number Variations (CNVs) have been analyzed by array comparative genomic hybridization (a-CGH)

Importantly, among 600 patients with molecular data included in the study, 51 (8.5%) patients operated on before 2009 presented with incomplete molecular data (especially regarding 1p19q co-deletion information). All of them benefitted from re-operation and were finally included in the study based on the molecular findings obtained during the second tissue sampling molecular analysis.

**Supplementary Methods 2.** Neuropsychological assessment

The neuropsychological assessment was performed by a neuropsychologist (G.H.) and a speech therapist (S.M.G). The following cognitive domains were evaluated: (1) Executive Functions, assessed with the part B and the part B minus A of the Trail Making Test (TMT), the STROOP test (subtest Interference and Interference minus Naming subtest), forward and backward digit span from the Wechsler Adult Intelligent Scale (WAIS-IV) and phonological and semantic verbal fluencies; (2) Psychomotor Speed and Attention assessed with the subtests “color naming” and “reading” from the STROOP task, and the part A of the TMT; (4) Visuospatial Function evaluated with the bell test (total omissions); (5) language and semantic abilities, evaluated with the DO80 picture naming task and the pyramid-and-palm-tree task (PPTT) (6) verbal episodic memory, assessed with the “Rappel libre et rappel indicé à 16 items (RL RI-16)”, and (7) Nonverbal Memory, examined with the immediate and delayed recall of the Rey-Osterrieth complex figure (RCF) or the Taylor complex figure (TCF). Note that, when available, alternate forms were used (*e.g.,* version a and b of the RLRI-16, ROCF was alternated with the TCF).

| **Neuropsychological domain** | **Neuropsychological tests** | **Cognitive abilities** | **Description** |
| --- | --- | --- | --- |
| Executives Functions | TMTB, TMT B-A | Cognitive flexibility, divided attention, visuomotor speed | Alternative switches between numbers and letters which had to be connected in ascending order, as rapidly as possible |
|  | STROOP Interference, STROOP I-D | Inhibition of competing response, selective attention, cognitive flexibility | Incongruent condition: the color-words are printed into an inconsistent color ink, the participant had to name the color and not to read |
|  | Forward span, Backward span | Immediate memory recall, verbal memory span, working memory | Immediate recall of a series of digits (forward and backward) |
|  | Phonological fluency | Cognitive flexibility, phonological thought | Producing words beginning by a given letter in a limited time |
|  | Categorical fluency | Cognitive flexibility, semantic memory | Producing words belonging to the same semantic categories (animals) in a limited time |
|  | TMT A | Visuomotor speed, attention | Connecting number in ascending order as rapidly as possible |
| Psychomotor Speed and Attention | STROOP naming | Psycho-motor speed, selective attention, | Congruent condition: naming color patches (congruous stimuli) as faster as possible |
|  | STROOP reading | Psycho-motor speed; selective attention | Congruent condition: read names of colors as faster as possible |
| Visuospatial function | Bell test: omissions | Visuospatial abilities, | target cancellation task, encircling bells among distractors |
| Language/semantics | Picture naming (DO80) | Naming (lexical retrieval) | Naming 80 black and white pictures |
|  | PPTT | Nonverbal semantic access, semantic representations | One target picture presented with two pictures; the patient is required to match one of both with the target one according to a semantic link |
|  |  |  |  |
| Verbal Episodic Memory | Free Recall 1, 2 and 3 and delayed free Recall (i.e. Free recall) | Verbal learning, organization of the verbal memory information, mid term verbal memory | Learning a list of 16 words, 3 immediate successive recalls divided in two parts: Free recall of the items in a limited time, |
|  | Recall 1, 2 and 3 and delayed Recall (i.e. Total recall) |  | 3 total recall in which categorical cues are given for the non-retrieval items in the free recall part, and |
|  |  |  | one delayed recall (20 minutes) followed by a total delayed recall. |
| Non-Verbal Memory | R&T: immediate and delayed recall | Visual memory, visuo-spatial integration, planning, organization | Reproducing the figure from memory (without forewarning) Immediately (3 minutes) and following a delay (30min) |

DO80: Dénomination orale d’images, PPTT: Pyramid and palm tree test, TMT: Trail making test, R&T Rey or Complex Figure.

Reference of the neurocognitive task: Trail Making Test, STROOP and verbal Fluencies,^1^ forward and backward digit span from the Wechsler Adult Intelligent Scale (WAIS IV),^2^ R&T: Rey or Taylor Complex Figure,^3–5^  the bell test,^6^ DO80: Dénomination orale d’images ^7^ , PPTT: Pyramid and palm tree test,^8^  RLRI16.^9^

1.Godefroy O. *Fonctions Exécutives et Pathologies Neurologiques et Psychiatriques: Évaluation En Pratique Clinique*. Groupe de Boeck; 2008.

2.Wechsler D. Wechsler adult intelligence scale–Fourth Edition (WAIS–IV). *San Antonio, TX: NCS Pearson*. 2008;22(498):1.

3.Rey A. L’examen psychologique dans les cas d’encéphalopathie traumatique.(Les problems.). *Archives de psychologie*. Published online 1941.

4.Osterrieth PA. Le test de copie d’une figure complexe; contribution a l’etude de la perception et de la memoire. *Archives de psychologie*. Published online 1944.

5.Taylor LB. Localisation of cerebral lesions by psychological testing. *Neurosurgery*. 1969;16(CN_suppl_1):269-287.

6.Gauthier L, Dehaut F, Joanette Y. The bells test: a quantitative and qualitative test for visual neglect. *International journal of clinical neuropsychology*. 1989;11(2):49-54.

7.Metz-Lutz MN. Standardisation d’un test de denomination orale: controle des effects de l’age, du sexe et du niveau de scolarite chez des sujets adultes normaux. *Rev Neuropsychol*. 1991;1:73-95.

8.Howard D, Patterson K. *The Pyramids and Palm Trees Test: A Test of Semantic Access from Words and Pictures*. Pearson Assessment; 1992.

9.Van der Linden M, Coyette F, Poitrenaud J, Kalafat M, Calicis F, Wyns C. L’épreuve de rappel libre/rappel indicé à 16 items (RL/RI-16)[Memory test assessment. Free recall with an index of 16 items (RL/RI-16)]. *LTévaluation des troubles de la mémoire Présentation de quatre tests de mémoire épisodique (avec leur étalonnage)*. Published online 2004.

**Supplementary Table 1.** Incidental findings: reasons for initial imaging

| **Reason for imaging** | **N (%, total=112)** |
| --- | --- |
| Headache | 50 (44.6) |
| Follow-up for other disease | 17 (15.2) |
| Research protocol | 2 (1.8) |
| Screening test for familial history | 1 (0.9) |
| ENT disorder | 26 (23.2) |
| Head trauma | 9 (8.0) |
| Other (meningitis, algodystrophy) | 7 (6.3) |

*ENT: ear, nose, throat*

**Supplementary Table 2.** Univariable Cox regression analyses (Overall survival, all cohort, n=600)

| **Variables** | **HR** | **95% CI** | ***p-value*** |
| --- | --- | --- | --- |
| Age at surgery | 0.98 | 0.96-1.00 | 0.109 |
| Oligo. vs. Astro | 0.28 | 0.17-0.44 | <0.0001 |
| Adjuvant chemotherapy | 1.41 | 0.55-2.98 | 0.417 |
| Supratotal vs partial resection | 0.08 | 0.00-0.40 | 0.015 |
| Total vs partial resection | 0.34 | 0.17-0.65 | 0.001 |
| Subtotal vs partial resection | 0.54 | 0.33-0.88 | 0.011 |

*Only variables complying with proportional hazard models assumptions were selected for analysis.*

*Astro.: IDH-mutant grade 2 astrocytoma, Oligo.: IDH-mutant, 1p19q codeleted grade 2 oligodendroglioma.*

**Supplementary Table 3.** Univariable Cox regression analyses (Overall survival with KPS≥80%, all cohort, n=600)

| **Variables** | **HR** | **95% CI** | ***p-value*** |
| --- | --- | --- | --- |
| Age at surgery | 0.99 | 0.97-1.01 | 0.227 |
| Oligo. vs. Astro | 0.27 | 1.16-3.52 | <0.0001 |
| Adjuvant chemotherapy | 2.10 | 0.55-2.98 | 0.009 |
| Supratotal vs partial resection | 0.13 | 0.03-0.36 | 0.001 |
| Total vs partial resection | 0.24 | 0.14-0.41 | <0.0001 |
| Subtotal vs partial resection | 0.45 | 0.31-0.65 | <0.0001 |

*Only variables complying with proportional hazard models assumptions were selected for analysis.*

*Astro.: IDH-mutant grade 2 astrocytoma, Oligo.: IDH-mutant, 1p19q codeleted grade 2 oligodendroglioma.*

**Supplementary Table 4.** Multivariable Cox regression analyses (Overall survival, all cohort, n=600)

| **Variables** | **HR** | **95% CI** | ***p-value*** |
| --- | --- | --- | --- |
| Age at surgery | 1.00 | 0.97-1.02 | 0.810 |
| Oligo. vs. Astro | 0.27 | 0.16-0.43 | <0.0001 |
| Adjuvant chemotherapy | 1.06 | 0.41-2.26 | 0.898 |
| Supratotal vs partial resection | 0.08 | 0.005-0.40 | 0.016 |
| Total vs partial resection | 0.31 | 0.16-0.59 | 0.0005 |
| Subtotal vs partial resection | 0.54 | 0.34-0.90 | 0.014 |

*Only variables complying with proportional hazard models assumptions were selected for analysis.*

*Astro.: IDH-mutant grade 2 astrocytoma, Oligo.: IDH-mutant, 1p19q codeleted grade 2 oligodendroglioma.*

**Supplementary Table 5.** Multiivariable Cox regression analyses (Overall survival with KPS≥80%, all cohort, n=600)

| **Variables** | **HR** | **95% CI** | ***p-value*** |
| --- | --- | --- | --- |
| Age at surgery | 0.99 | 0.97-1.01 | 0.227 |
| Oligo. vs. Astro | 0.27 | 1.16-3.52 | <0.0001 |
| Adjuvant chemotherapy | 2.10 | 0.55-2.98 | 0.009 |
| Supratotal vs partial resection | 0.13 | 0.03-0.36 | 0.001 |
| Total vs partial resection | 0.24 | 0.14-0.41 | <0.0001 |
| Subtotal vs partial resection | 0.45 | 0.31-0.65 | <0.0001 |

*Only variables complying with proportional hazard models assumptions were selected for analysis.*

*Astro.: IDH-mutant grade 2 astrocytoma, Oligo.: IDH-mutant, 1p19q codeleted grade 2 oligodendroglioma.*

**Supplementary Table 6.** Characteristics of patients stratified by risk group, based on overall survival prognosis as determined by recursive partitioning analysis (RPA).

| **Variables** | **Low risk (n=318)** | **Intermediate risk (n=230)** | **High risk (n=52)** | ***p-value*** |
| --- | --- | --- | --- | --- |
| Age at surgery, years, median (IQR) | 37 (31-45) | 34 (29-41) | 32 (26-38) | 0.0002^a^ |
| *Sex* |  |  |  |  |
| Female, n (%) | 156 (49.1) | 96 (41.7) | 24 (46.2) | 0.275^b^ |
| Male, n (%) | 162 (50.9) | 134 (58.3) | 28 (53.8) |  |
| *Hstomolecular status* |  |  |  |  |
| Astrocytoma, n (%) | 119 (37.4) | 164 (71.3) | 52 (100.0) | <0.0001^b^ |
| 1p19q codeleted oligodendroglioma, n (%) | 199 (62.6) | 66 (28.7) | 0 (0.0) |  |
| *Tumor location* |  |  |  |  |
| Left hemisphere, n (%) | 192 (60.4) | 131 (57.0) | 30 (57.7) | 0.230^b^ |
| Right hemisphere, n (%) | 125 (39.3) | 94 (40.9) | 22 (42.3) |  |
| Bilateral, n (%) | 1 (0.3) | 5 (2.2) | 0 (0.0) |  |
| *Tumor volumes* |  |  |  |  |
| Presurgical TV, mL, median (IQR) | 26.0 (14-50.0) | 50 (23-92) | 130 (100.0-170.0) | <0.0001^a^ |
| Postsurgical TV, mL, median (IQR) | 0.5 (0.0-3.0) | 2.5 (0.0-10.0) | 23.0 (17.0-29.5) | <0.0001^a^ |
| *Preoperative seizures* |  |  |  |  |
| Yes, n (%) | 232 (73.0) | 200 (87.0) | 50 (96.2) | <0.0001^b^ |
| No, n (%) | 86 (27.0) | 30 (13.0) | 2 (3.8) |  |
| *Postoperative seizures* |  |  |  |  |
| Long term (>3 months), n (%) | 11 (3.5) | 24 (10.4) | 8 (15.4) | 0.0004^b^ |
| Transient (<3 months), n (%) | 27 (8.5) | 20 (8.7) | 7 (13.5) | 0.499^b^ |
| *Adjuvant chemotherapy* |  |  |  |  |
| Yes, n (%) | 15 (7.7) | 18 (7.8) | 13 (25.0) | <0.0001^b^ |
| No, n (%) | 303 (92.3) | 212 (92.2) | 39 (75.0) |  |
| *Adjuvant radiotherapy* |  |  |  |  |
| Yes, n (%) | 3 (1.5) | 3 (1.3) | 3 (5.7) | 0.0281^b^ |
| No, n (%) | 315 (98.5) | 227 (98.7) | 49 (94.2) |  |
| Extent of resection (median, IQR) | 98.1 (92.0-100.0) | 95.0 (88.0-100.0) | 82.0 (79.0-86.0) | <0.0001^a^ |
| *Type of resection* |  |  |  |  |
| Supratotal, n (%) | 47 (14.8) | 2 (0.9) | 0 (0.0) | <0.0001^b^ |
| Total, n (%) | 109 (34.3) | 25 (10.9) | 0 (0.0) | <0.0001^b^ |
| Subtotal, n (%) | 162 (50.9) | 141 (61.3) | 0 (0.0) | <0.0001^b^ |
| Partial, n (%) | 0 (0.0) | 62 (27.0) | 52 (100.0) | <0.0001^b^ |
| *Karnofsky performance status* |  |  |  |  |
| Preoperative KPS (median, IQR) | 100 (90-100) | 100 (90-100) | 90 (90-100) | <0.0001^a^ |
| Postoperative KPS, 3 months (median, IQR) | 100 (90-100) | 90 (90-100) | 90 (80-95) | <0.0001^a^ |
| Postoperative deficit, n (%) | 1 (0.3) | 3 (1.3) | 0 (0.0) | 0.307^b^ |
| *Professional activities* |  |  |  |  |
| Active before surgery, n (%) | 280 (88.1) | 185 (80.4) | 39 (75.0) | 0.010^b^ |
| Active after surgery, n (%) | 268 (84.3) | 173 (75.2) | 31 (59.6) | <0.0001^b^ |
|  |  |  |  |  |

a Kruskal-Wallis test

b Chi-squared test

**Supplementary Table 7.** Characteristics of patients stratified by risk group, based on overall survival with KPS≥80% prognosis as determined by RPA.

| **Variables** | **Low risk (n=192)** | **Intermediate risk (n=322)** | **High risk (n=86)** | ***p-value*** |
| --- | --- | --- | --- | --- |
| Age at surgery, years, median (IQR) | 39 (32.25-47) | 36 (29-42) | 32.5 (29-38.75) | <0.0001^a^ |
| *Sex* |  |  |  |  |
| Female, n (%) | 94 (49.0) | 148 (46.0) | 34 (39.5) | 0.346^b^ |
| Male, n (%) | 98 (51.0) | 174 (54.0) | 52 (60.5) |  |
| *Hstomolecular status* |  |  |  |  |
| Astrocytoma, n (%) | 0 (0.0) | 249 (77.3) | 86 (100.0) | <0.0001^b^ |
| 1p19q codeleted oligodendroglioma, n (%) | 192 (100.0) | 73 (22.7) | 0 (0.0) |  |
| *Tumor location* |  |  |  |  |
| Left hemisphere, n (%) | 100 (52.1) | 202 (62.7) | 51 (59.3) | 0.069^b^ |
| Right hemisphere, n (%) | 91 (47.4) | 115 (35.7) | 35 (40.7) |  |
| Bilateral, n (%) | 1 (0.5) | 5 (1.6) | 0 (0.0) |  |
| *Tumor volumes* |  |  |  |  |
| Presurgical TV, mL, median (IQR) | 37.0 (19-65.0) | 35.0 (20-62) | 121.5 (105.0-159.8) | <0.0001^a^ |
| Postsurgical TV, mL, median (IQR) | 1.0 (0.0-4.0) | 2.05 (0.0-7.0) | 14.0 (9.0-23.75) | <0.0001^a^ |
| *Preoperative seizures* |  |  |  |  |
| Yes, n (%) | 152 (79.2) | 250 (77.6) | 80 (93.0) | 0.0055^b^ |
| No, n (%) | 40 (20.8) | 72 (22.4) | 6 (7.0) |  |
| *Postoperative seizures* |  |  |  |  |
| Long term (>3 months), n (%) | 4 (2.1) | 33 (10.2) | 6 (7.0) | 0.0024^b^ |
| Transient (<3 months), n (%) | 11 (5.7) | 39 (12.1) | 4 (4.7) | 0.0158^b^ |
| *Adjuvant chemotherapy* |  |  |  |  |
| Yes, n (%) | 11 (5.7) | 18 (5.6) | 17 (19.8) | <0.0001^b^ |
| No, n (%) | 181 (94.3) | 304 (94.4) | 69 (80.2) |  |
| *Adjuvant radiotherapy* |  |  |  |  |
| Yes, n (%) | 4 (2.1) | 3 (0.9) | 2 (2.3) | 0.462^b^ |
| No, n (%) | 188 (97.9) | 319 (99.1) | 84 (97.7) |  |
| Extent of resection (median, IQR) | 97 (93.0-100.0) | 94.0 (85.0-100.0) | 89.0 (83.50-92.75) | <0.0001^a^ |
| *Type of resection* |  |  |  |  |
| Supratotal, n (%) | 22 (11.5) | 27 (8.4) | 0 (0.0) | 0.0054^b^ |
| Total, n (%) | 54 (28.1) | 77 (23.9) | 3 (3.5) | <0.0001^b^ |
| Subtotal, n (%) | 110 (57.3) | 169 (52.5) | 24 (27.9) | <0.0001^b^ |
| Partial, n (%) | 6 (3.1) | 49 (15.2) | 59 (68.6) | <0.0001^b^ |
| *Karnofsky performance status* |  |  |  |  |
| Preoperative KPS (median, IQR) | 100 (90-100) | 100 (90-100) | 90 (90-100) | 0.0016^a^ |
| Postoperative KPS, 3 months (median, IQR) | 100 (90-100) | 90 (90-100) | 90 (90-100) | 0.0048^a^ |
| Postoperative deficit, n (%) | 1 (0.5) | 3 (0.9) | 0 (0.0) | 0.613^b^ |
| *Professional activities* |  |  |  |  |
| Active before surgery, n (%) | 156 (81.3) | 276 (85.7) | 72 (83.7) | 0.409^b^ |
| Active after surgery, n (%) | 149 (77.6) | 261 (81.1) | 62 (72.1) | 0.179^b^ |
|  |  |  |  |  |

a Kruskal-Wallis test

b Chi-squared test

**Supplementary Table 8.** Univariable Cox regression analyses (risk groups based on overall survival prognosis as determined by RPA).

| **Variables** | **HR** | **95% CI** | ***p-value*** |
| --- | --- | --- | --- |
| Low risk vs High risk | 0.18 | 0.10-0.35 | <0.0001 |
| Low risk vs Intermediate risk | 0.44 | 0.27-0.70 | 0.0007 |
| Intermediate risk vs High risk | 0.42 | 0.25-0.76 | 0.0026 |
|  |  |  |  |

**Supplementary Table 9.** Univariable Cox regression analyses (risk groups based on overall survival with KPS≥80% prognosis as determined by RPA).

| **Variables** | **HR** | **95% CI** | ***p-value*** |
| --- | --- | --- | --- |
| Low risk vs High risk | 0.60 | 0.43-0.86 | 0.0038 |
| Low risk vs Intermediate risk | 0.73 | 0.59-0.89 | 0.0027 |
| Intermediate risk vs High risk | 0.82 | 0.60-1.15 | 0.0234 |
|  |  |  |  |

**Supplementary Table 10.** Summary of the balance of matched data

|  | **Standardized mean difference of propensity score measure** | **Standardized pair distance** |
| --- | --- | --- |
| Supratotal vs non-supratotal (1:3) | 0.048 | 0.043 |
| Supratotal vs non-supratotal (1:2) | 0.031 | 0.026 |
| Supratotal vs non-supratotal (1:1) | 0.071 | 0.074 |
| Total vs less than Total resection (1:1) | 0.118 | 0.113 |
| Supratotal vs Total (1:1) | 0.050 | 0.063 |
| Supratotal vs Total (1:2) | 0.059 | 0.065 |
|  |  |  |

The matching process was performed with the MatchIt package (https://cran.r-project.org/web/packages/MatchIt) and the optmatch package (https://cran.r-project.org/web/packages/optmatch).

**Supplementary Table 11.** Characteristics of patients selected by propensity score matching (supraTR vs. TR, ratio of 1:2).

| **Variables** | **supraTR**  **(n=46)** | **TR**  **(n=67)** | ***p-value*** |
| --- | --- | --- | --- |
| Age at surgery, years, median (IQR) | 36 (31-49) | 37 (32-48) | 0.719^a^ |
| *Sex* |  |  |  |
| Female, n (%) | 23 (50.0) | 35 (52.2) | 0.850^b^ |
| Male, n (%) | 23 (50.0) | 32 (47.8) |  |
| *Hstomolecular status* |  |  |  |
| Astrocytoma, n (%) | 27 (58.7) | 39 (58.2) | >0.999^b^ |
| 1p19q codeleted oligodendroglioma, n (%) | 19 (41.3) | 28 (41.8) |  |
| *Tumor location* |  |  |  |
| Left hemisphere, n (%) | 28 (60.9) | 37 (55.2) | 0.568^b^ |
| Right hemisphere, n (%) | 18 (39.1) | 30 (44.8) |  |
| *Tumor volumes* |  |  |  |
| Presurgical TV, mL, median (IQR) | 13 (5.35-25.50) | 15 (10-24.0) | 0.234^a^ |
| *Preoperative seizures* |  |  |  |
| Yes, n (%) | 21 (45.7) | 33 (49.3) | 0.848^b^ |
| No, n (%) | 25 (54.3) | 34 (50.7) |  |
| *Adjuvant chemotherapy* |  |  |  |
| Yes, n (%) | 1 (2.2) | 1 (1.5) | >0.999^b^ |
| No, n (%) | 45 (97.8) | 66 (98.5) |  |
| *Adjuvant radiotherapy* |  |  |  |
| Yes, n (%) | 0 (0.0) | 0 (0.0) | >0.999^b^ |
| No, n (%) | 46 (100.0) | 67 (100.0) |  |
| *Karnofsky performance status* |  |  |  |
| Preoperative KPS (median, IQR) | 100 (100-100) | 100 (100-100) | 0.333^a^ |
| Postoperative KPS, 3 months (median, IQR) | 100 (100-100) | 100 (90-100) | 0.478^a^ |
| Postoperative deficit, n (%) | 0 (0.0) | 0 (0.0) | >0.999^b^ |
| *Professional activities* |  |  |  |
| Active before surgery, n (%) | 40 (87.0) | 60 (89.6) | 0.767^b^ |
| Active after surgery, n (%) | 38 (82.6) | 57 (85.1) | 0.375^b^ |
|  |  |  |  |

^a^ two-tailed Mann-Whitney U test.

^b^ Fisher’s exact test

**Supplementary Table 12.** Characteristics of patients selected by propensity score matching (supraTR vs. non-supraTR, ratio of 1:3).

| **Variables** | **supraTR**  **(n=42)** | **non-supraTR**  **(n=95)** | ***p-value*** |
| --- | --- | --- | --- |
| Age at surgery, years, median (IQR) | 37 (32.75-49) | 40 (31-47) | 0.850^a^ |
| *Sex* |  |  |  |
| Female, n (%) | 21 (50.0) | 46 (48.4) | >0.999^b^ |
| Male, n (%) | 21 (50.0) | 49 (51.6) |  |
| *Hstomolecular status* |  |  |  |
| Astrocytoma, n (%) | 24 (57.1) | 54 (56.8) | >0.999^b^ |
| 1p19q codeleted oligodendroglioma, n (%) | 18 (42.9) | 41 (43.2) |  |
| *Tumor location* |  |  |  |
| Left hemisphere, n (%) | 25 (59.5) | 57 (60.0) | >0.999^b^ |
| Right hemisphere, n (%) | 17 (40.5) | 38 (40.0) |  |
| *Tumor volumes* |  |  |  |
| Presurgical TV, mL, median (IQR) | 15.5 (6-25.25) | 16 (10.0-26.0) | 0.199^a^ |
| *Preoperative seizures* |  |  |  |
| Yes, n (%) | 19 (45.2) | 46 (48.4) | 0.853^b^ |
| No, n (%) | 23 (54.8) | 49 (51.6) |  |
| *Adjuvant chemotherapy* |  |  |  |
| Yes, n (%) | 1 (2.4) | 3 (3.2) | >0.999^b^ |
| No, n (%) | 41 (97.6) | 92 (96.8) |  |
| *Adjuvant radiotherapy* |  |  |  |
| Yes, n (%) | 0 (0.0) | 0 (0.0) | >0.999^b^ |
| No, n (%) | 42 (100.0) | 95 (100.0) |  |
| *Karnofsky performance status* |  |  |  |
| Preoperative KPS (median, IQR) | 100 (100-100) | 100 (100-100) | 0.432^a^ |
| Postoperative KPS, 3 months (median, IQR) | 100 (90-100) | 100 (90-100) | 0.556^a^ |
| Postoperative deficit, n (%) | 0 (0.0) | 0 (0.0) | >0.999^b^ |
| *Professional activities* |  |  |  |
| Active before surgery, n (%) | 36 (85.7) | 86 (90.5) | 0.392^b^ |
| Active after surgery, n (%) | 34 (81.0) | 80 (84.2) | 0.628^b^ |
|  |  |  |  |

^a^ two-tailed Mann-Whitney U test.

^b^ Fisher’s exact test

**Supplementary Table 13.** Characteristics of patients selected by propensity score matching (TR+ vs. TR-, ratio of 1:1).

| **Variables** | **TR+**  **(n=142)** | **TR-**  **(n=142)** | ***p-value*** |
| --- | --- | --- | --- |
| Age at surgery, years, median (IQR) | 36.5 (30-43.25) | 36.5 (30-44.25) | 0.859^a^ |
| *Sex* |  |  |  |
| Female, n (%) | 69 (48.6) | 74 (52.1) | 0.635^b^ |
| Male, n (%) | 73 (51.4) | 68 (47.9) |  |
| *Hstomolecular status* |  |  |  |
| Astrocytoma, n (%) | 79 (55.6) | 77 (54.2) | 0.905^b^ |
| 1p19q codeleted oligodendroglioma, n (%) | 63 (44.4) | 65 (45.8) |  |
| *Tumor location* |  |  |  |
| Left hemisphere, n (%) | 78 (54.9) | 79 (55.6) | >0.999^b^ |
| Right hemisphere, n (%) | 64 (45.1) | 63 (44.4) |  |
| *Tumor volumes* |  |  |  |
| Presurgical TV, mL, median (IQR) | 25.0 (14.75-36.50) | 27.0 (16.0-39.0) | 0.168^a^ |
| *Type of resection* |  |  |  |
| Supratotal, n (%) | 34 (23.9) | 0 (0.0) | <0.0001^b^ |
| Total, n (%) | 108 (76.1) | 0 (0.0) | <0.0001^b^ |
| *Preoperative seizures* |  |  |  |
| Yes, n (%) | 97 (68.3) | 105 (73.9) | 0.359^b^ |
| No, n (%) | 45 (31.7) | 37 (26.1) |  |
| *Adjuvant chemotherapy* |  |  |  |
| Yes, n (%) | 5 (3.5) | 4 (2.8) | >0.999^b^ |
| No, n (%) | 137 (96.5) | 138 (97.2) |  |
| *Adjuvant radiotherapy* |  |  |  |
| Yes, n (%) | 1 (0.7) | 0 (0.0) | >0.999^b^ |
| No, n (%) | 141 (99.3) | 142 (100.0) |  |
| *Karnofsky performance status* |  |  |  |
| Preoperative KPS (median, IQR) | 100 (90-100) | 100 (90-100) | 0.933^a^ |
| Postoperative KPS, 3 months (median, IQR) | 100 (90-100) | 100 (90-100) | 0.575^a^ |
| Postoperative deficit, n (%) | 0 (0.0) | 1 (0.7) | >0.999^b^ |
| *Professional activities* |  |  |  |
| Active before surgery, n (%) | 124 (87.3) | 126 (88.7) | 0.855^b^ |
| Active after surgery, n (%) | 119 (83.8) | 124 (87.3) | 0.509^b^ |
|  |  |  |  |

^a^ two-tailed Mann-Whitney U test.

^b^ Fisher’s exact test

TR+: at least total resection (extent of surgical resection ≥100%), TR-: less than total resection (extent of surgical resection <100%)

**Supplementary Table 14.** Univariable Cox regression analyses (Overall survival comparisons, as determined by propensity score matching).

| **Variables** | **HR** | **95% CI** | ***p-value*** |
| --- | --- | --- | --- |
| SupraTR^a^ vs non-supraTR  SupraTR^b^ vs TR  TR+ vs TR- | undefined*  undefined*  0.41 | undefined*  undefined*  0.19-0.84 | undefined*  undefined*  0.0188 |
|  |  |  |  |

*supraTR^a^ group comprised 42 subjects*

*non-supraTR group comprised 95 subjects*

*supraTb^a^ group comprised 46 subjects*

*TR group comprised 67 subjects*

*TR+ group comprised 142 subjects*

*TR- group comprised 142 subjects*

** Proportional hazard models assumptions were not met*

**Supplementary Figure 1.** Geographical distribution of patients included in the cohort.

**
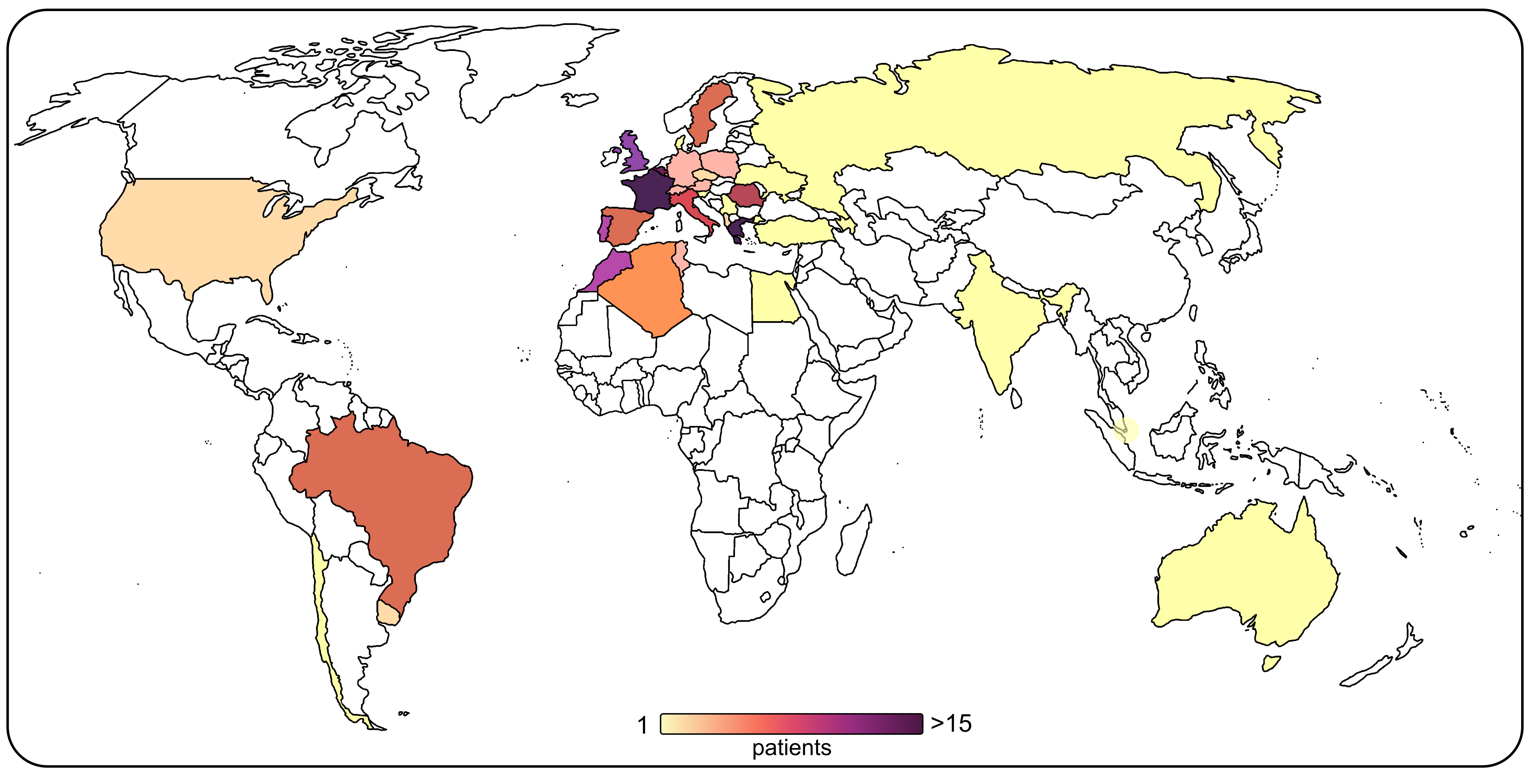
**

Among 600 patients selected for analysis, 464 patients (77.3%) were French residents, 136 were foreigners (22.6%), and came from 34 countries, speaking 20 different languages.

**Supplementary Figure 2.** Kaplan-Meier curves and Hazard ratios for Progression-free survival.





A. Kaplan-Meier curves for progression-free survival (PFS) from initial surgery in all patients (n=600). B. Hazard ratio results for PFS (n=600) using multivariate Cox proportional hazard models in variables eligible for analyses.

**Supplementary Figure 3.** Survival curves for supraTR+ vs. non-supraTR, as determined by propensity score matching with a ratio of 1:1.


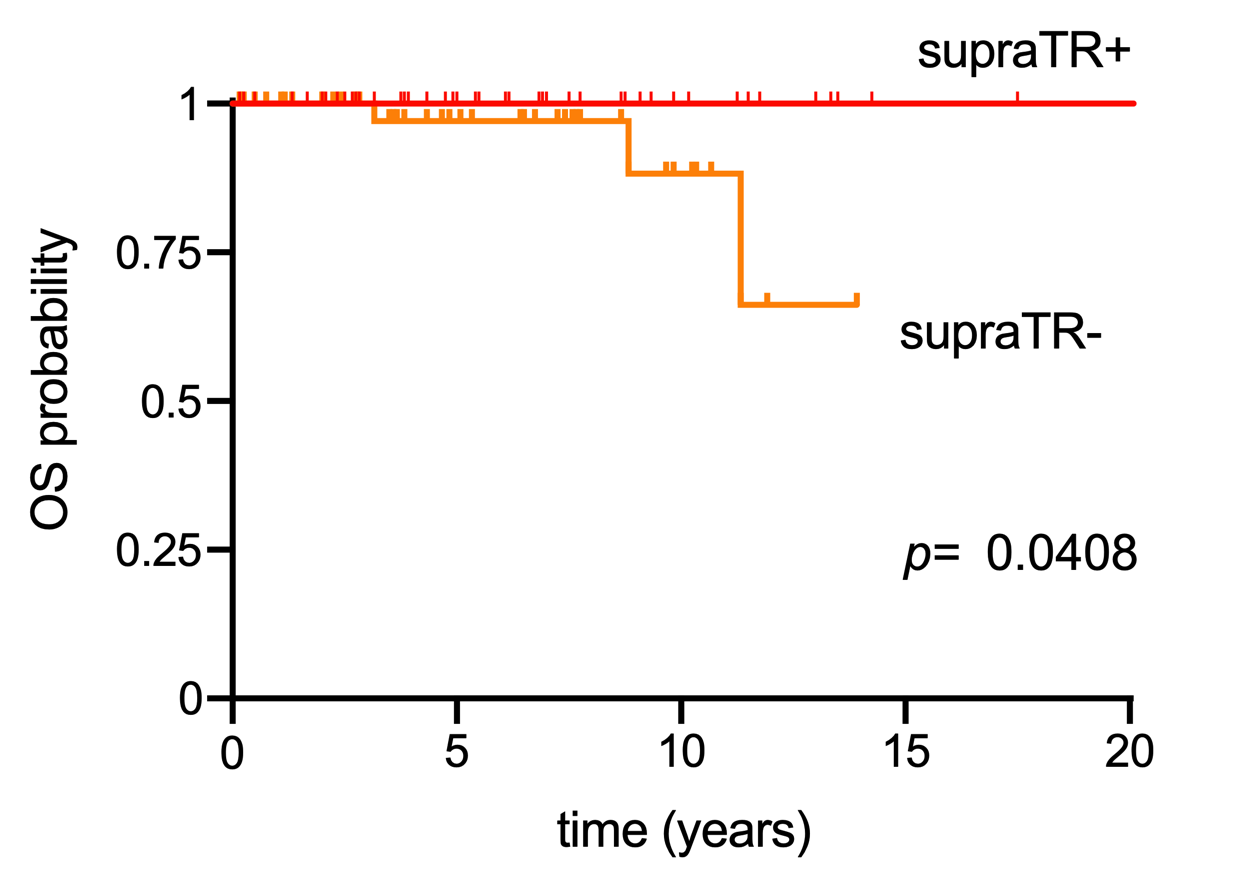


*P-value was determined by Log-rank test*

*supraTR+ group comprised 49 subjects*

*supraTR- group comprised 46 subjects*

**Supplementary Figure 4.** Survival curves for supraTR+ vs. non-supraTR, as determined by propensity score matching with a ratio of 1:2.


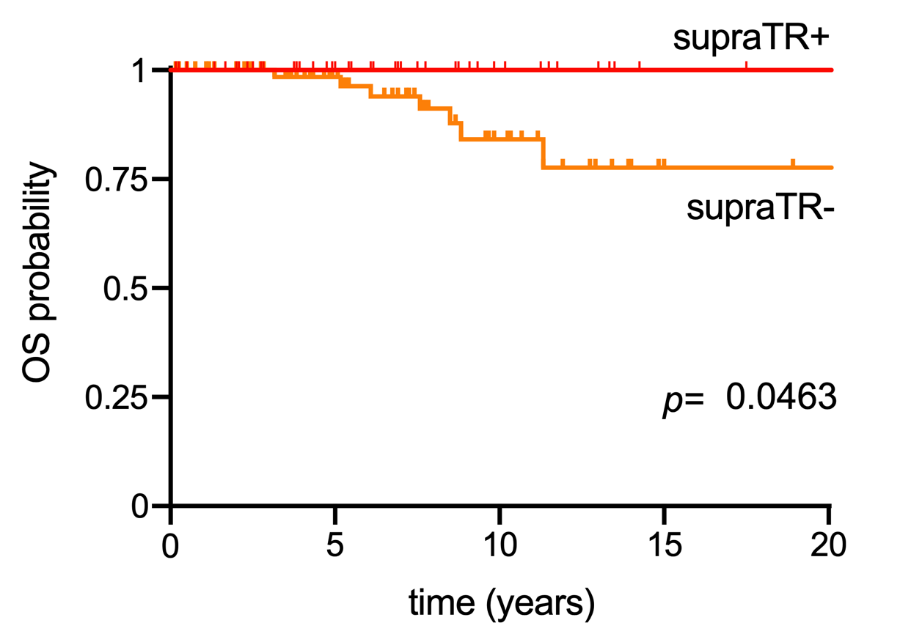


*P-value was determined by Log-rank test*

*supraTR+ group comprised 46 subjects*

*supraTR- group comprised 72 subjects*

**Supplementary Figure 5.** Survival curves for supraTR+ vs. non-supraTR, as determined by propensity score matching with a ratio of 1:3.

*
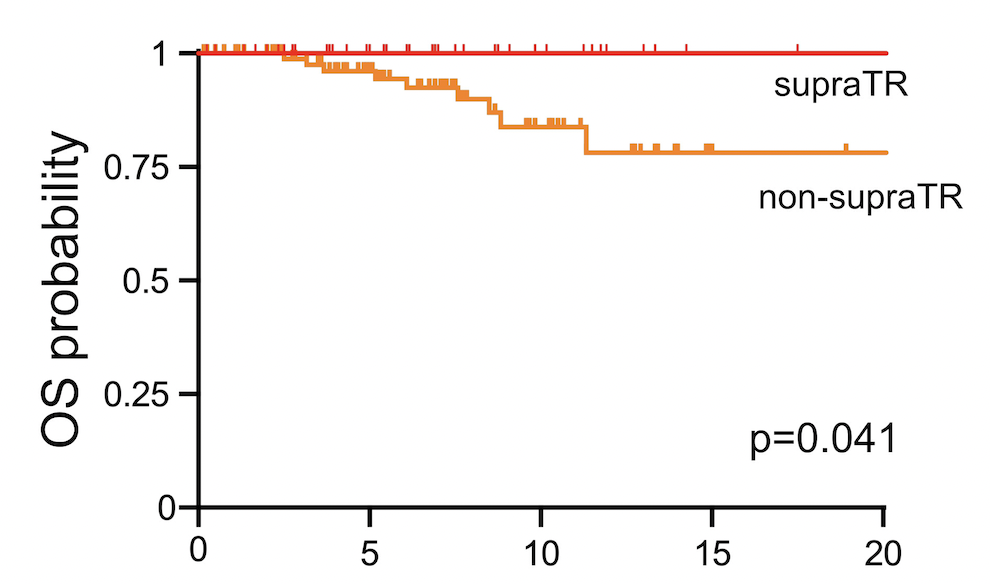
*

time (years)

*P-value was determined by Log-rank test*

*supraTR+ group comprised 42 subjects*

*supraTR- group comprised 95 subjects*

**Supplementary Figure 6.** Survival curves for supraTR vs. TR, as determined by propensity score matching with a ratio of 1:1.


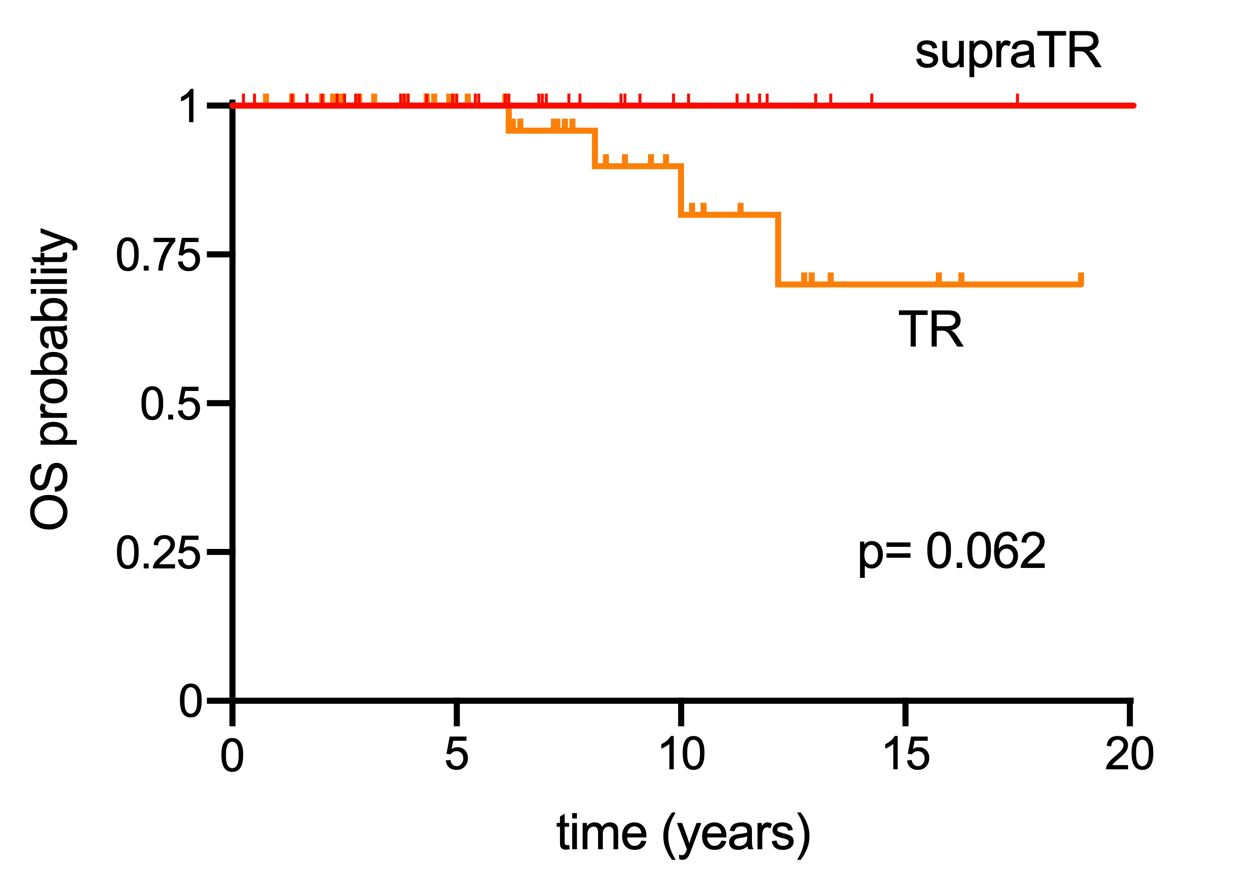


*P-value was determined by Log-rank test*

*supraTR group comprised 42 subjects*

*TR group comprised 42 subjects*

**Supplementary Figure 7**

Progression-free survival curves for supraTR vs. TR, as determined by propensity score matching with a ratio of 1:1.


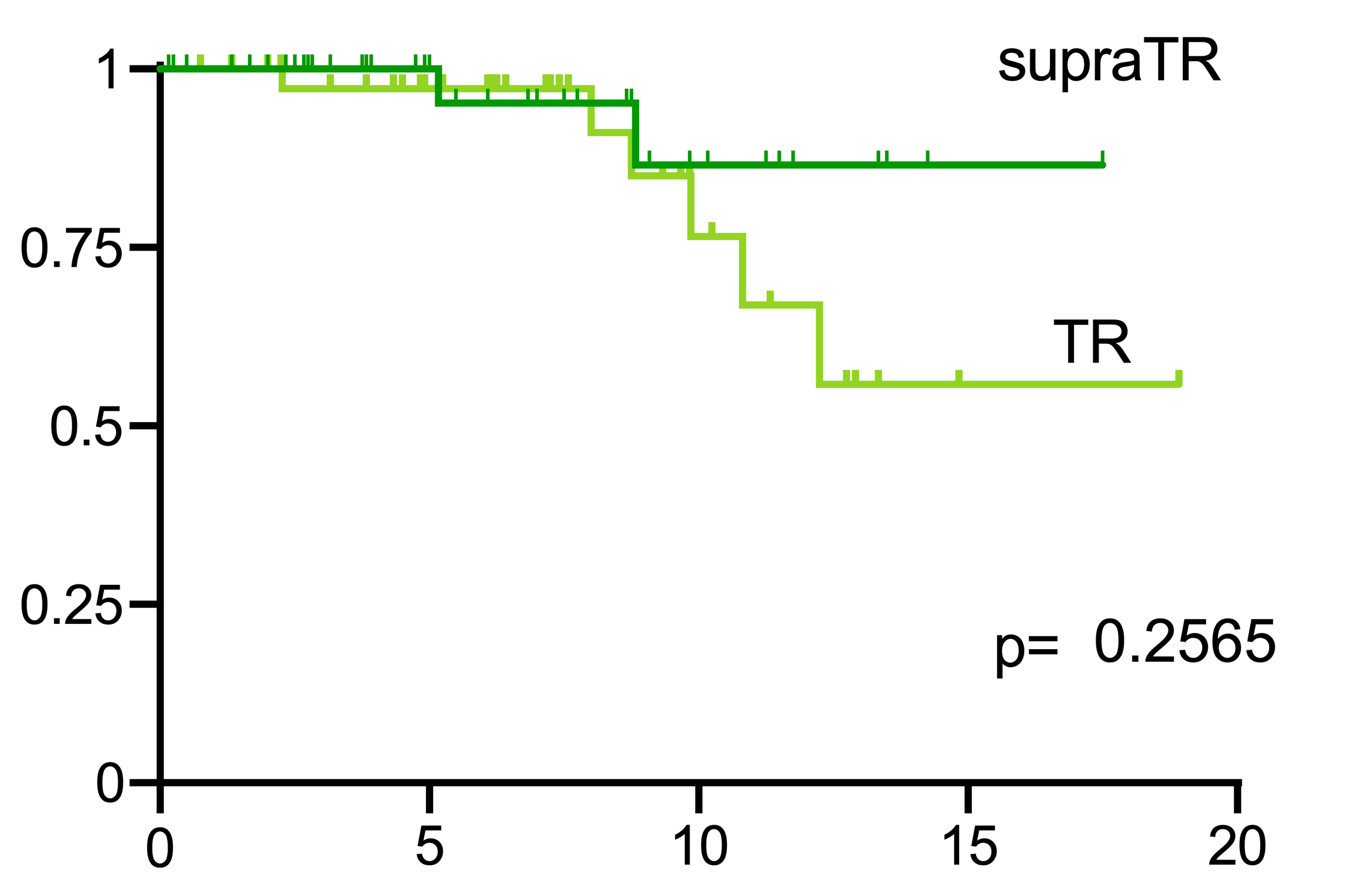


*P-value was determined by Log-rank test*

*supraTR group comprised 42 subjects*

*TR group comprised 42 subjects*

**Supplementary Figure 8**

Progression-free survival curves for supraTR vs. TR, as determined by propensity score matching with a ratio of 1:2.


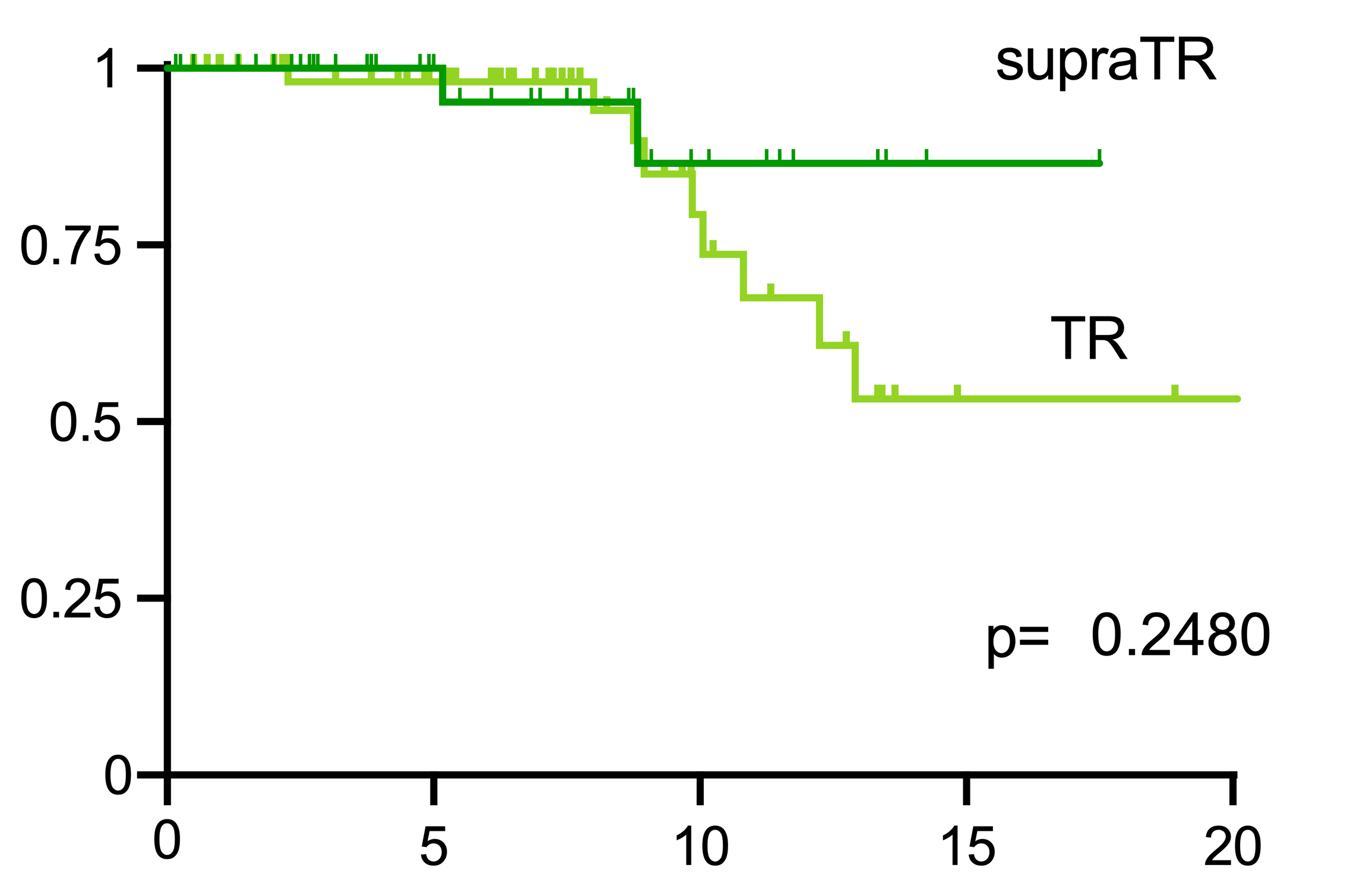


*P-value was determined by Log-rank test*

*supraTR group comprised 46 subjects*

*TR group comprised 67 subjects*

**Supplementary Figure 9**

Propensity score analysis of overall survival in patients with supratotal resection (supraTR) vs. patients with total resection (TR), by preoperative tumore volume (mL).


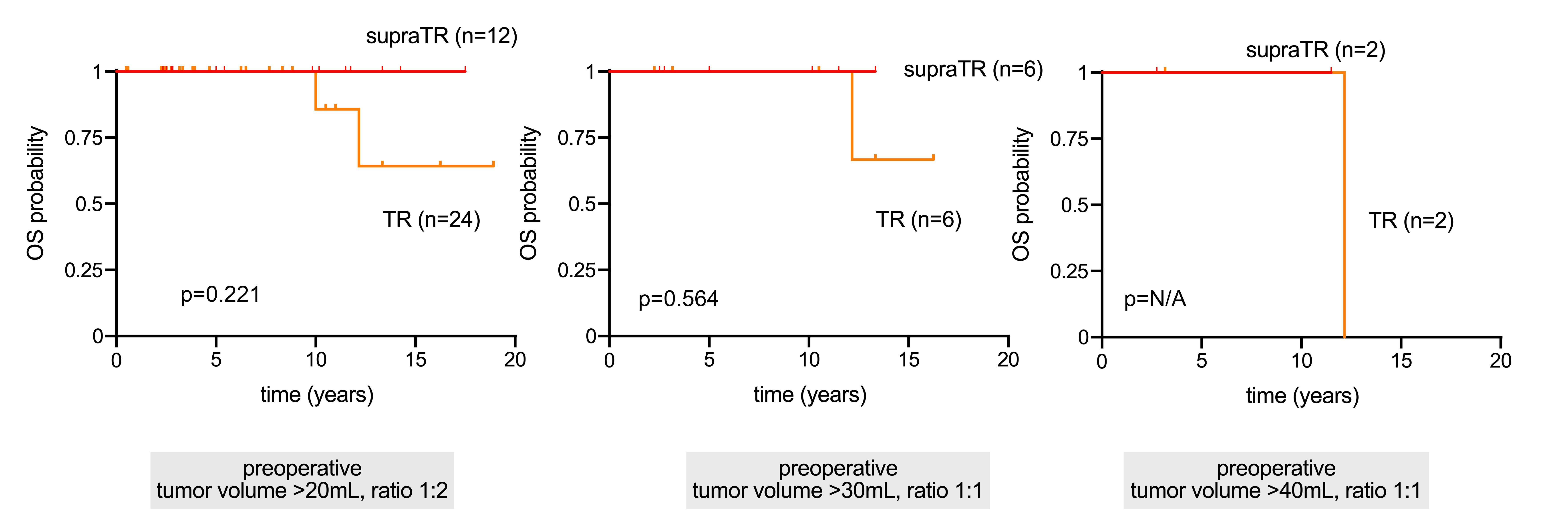


From left to right, Kaplan-Meier curves for overall survival in supraTR group (n=12) vs TR group (n=24, propensity matching, ratio 1:2) in patients with preoperative tumor volume >20mL, Kaplan-Meier curves for overall survival in supraTR group (n=6) vs TR group (n=6, propensity matching, ratio 1:1) in patients with preoperative tumor volume >30mL and Kaplan-Meier curves for overall survival in supraTR group (n=2) vs TR group (n=2, propensity matching, ratio 1:1) in patients with preoperative tumor volume >40mL. Log-rank tests were used for statistical comparisons. N/A: not applicable.
